# Genomic variation and DNA repair associated with soybean transgenesis: a comparison to cultivars and mutagenized plants

**DOI:** 10.1186/s12896-016-0271-z

**Published:** 2016-05-12

**Authors:** Justin E. Anderson, Jean-Michel Michno, Thomas J. Y. Kono, Adrian O. Stec, Benjamin W. Campbell, Shaun J. Curtin, Robert M. Stupar

**Affiliations:** Department of Agronomy & Plant Genetics, University of Minnesota, 1991 Upper Buford Circle, 411 Borlaug Hall, St. Paul, MN 55108 USA

**Keywords:** Somaclonal variation, Structural variation, Genetic engineering, Biotechnology, Transgenic crops, Soybean

## Abstract

**Background:**

The safety of mutagenized and genetically transformed plants remains a subject of scrutiny. Data gathered and communicated on the phenotypic and molecular variation induced by gene transfer technologies will provide a scientific-based means to rationally address such concerns. In this study, genomic structural variation (e.g. large deletions and duplications) and single nucleotide polymorphism rates were assessed among a sample of soybean cultivars, fast neutron-derived mutants, and five genetically transformed plants developed through *Agrobacterium* based transformation methods.

**Results:**

On average, the number of genes affected by structural variations in transgenic plants was one order of magnitude less than that of fast neutron mutants and two orders of magnitude less than the rates observed between cultivars. Structural variants in transgenic plants, while rare, occurred adjacent to the transgenes, and at unlinked loci on different chromosomes. DNA repair junctions at both transgenic and unlinked sites were consistent with sequence microhomology across breakpoints. The single nucleotide substitution rates were modest in both fast neutron and transformed plants, exhibiting fewer than 100 substitutions genome-wide, while inter-cultivar comparisons identified over one-million single nucleotide polymorphisms.

**Conclusions:**

Overall, these patterns provide a fresh perspective on the genomic variation associated with high-energy induced mutagenesis and genetically transformed plants. The genetic transformation process infrequently results in novel genetic variation and these rare events are analogous to genetic variants occurring spontaneously, already present in the existing germplasm, or induced through other types of mutagenesis. It remains unclear how broadly these results can be applied to other crops or transformation methods.

**Electronic supplementary material:**

The online version of this article (doi:10.1186/s12896-016-0271-z) contains supplementary material, which is available to authorized users.

## Background

Plant breeders use genetic variation from elite and diverse lines as the primary source for cultivar development and trait improvement. In some cases, traits of interest cannot be found within this “standing” variation in the current germplasm. However, mutagenesis or genetic transformation can provide a means to introduce such traits. Standard mutagenesis treatments, such as Fast Neutron (FN) irradiation, alter DNA sequences at random loci throughout the genome in an attempt to generate novel trait variation [1]. Genetic transformation, alternatively, attempts to insert one or few transgenes to confer a novel trait or disrupt the activity of an endogenous gene.

The genetic transformation of most crop species requires plant tissue culture methods, which can introduce heritable phenotypes caused by unintended genetic and epigenetic changes [2]. These unintended changes, known as somaclonal variation, may theoretically compromise the safety of transgenic plants [3]. Therefore, it is important to understand the coupled effects of genetic transformation and tissue culture [4] and how these compare to standing and other types of induced variation.

Naturally occurring variation is a well-established source of novel phenotypes in many vegetatively propagated fruits and vegetables, where they are commonly known as ‘sports’ [5]. Somaclonal variation induced through tissue culture, first observed in sugarcane (*Saccharum*) [6], has been reported in many other plant species [2]. Desirable agronomic traits and released cultivars have even been derived from this type of induced variation [7]. The molecular underpinnings of somaclonal variation can include DNA sequence changes, chromosome rearrangements, aneuploidy, activation of transposable elements, and epigenetic restructuring [2]. Genome-wide single nucleotide changes resulting from tissue culture have been recently observed using next-generation sequencing (NGS) in Arabidopsis [8] and rice [9–13]. These studies suggest tissue culture might increase the single nucleotide mutation rate and may activate transposons [14].

The insertion of a transgene is also known to create localized or dispersed genomic changes. Recent studies found that transformation can result in DNA inserted at multiple loci, multiple transgenes per locus, fragmented T-DNA, and chromosome rearrangements [15–19], though such complex events are rare and often discarded rather than commercialized. In Arabidopsis, transgene insertion is generally random across chromosomes, in both genic and non-genic sequences, and frequently associated with a deletion ranging from 11 to 100 bp in size [20]. For soybean (*Glycine max)*, *Agrobacterium* based transformation methods occasionally result in multiple insertion sites, tandem insertions, and integration of plasmid backbone sequences [21]. Recently, resequencing methods have been used to accurately localize and resolve transgene insertions in different plant species [19, 22–24]. While advanced technologies have helped detect the local and dispersed effects of tissue culture and transformation, limitations still exist due to sequencing errors, genetic heterogeneity of plant accessions, and reference bias [25].

Separating the changes induced by transformation from pre-existing genetic variation can be a challenge [26]. Plant genomes can vary dramatically between cultivars. A large portion of this variation occurs as genomic structural variants (SV), such as large deletions and duplications [27]. These SV are associated with a number of biologically and agriculturally important traits [27]. Previous studies in soybean have used array-based comparative genomic hybridization (CGH) or resequencing approaches to observe levels of standing SV among accessions [28, 29], or SV induced through FN mutagenesis [1]. However, no comparable studies have addressed the incidence of tissue culture and transformation on rates of genome-wide SV in soybean.

This study investigates five transgenic (T_1_ generation) soybean plants derived from standard *Agrobacterium*-mediated transformation. SV in these five plants was assessed by CGH and two of these plants were resequenced to ascertain the frequency of nucleotide substitutions. These data allow for comparisons of genomic variation in transgenic plants to the genomic variation observed in mutagenized and standing accessions. These analyses provide new insight towards understanding somaclonal variation, the effects of transgene insertion, the inheritance of SV, and the genomic consequences of developing mutant and transgenic stocks as compared to the standing variation already present in the soybean germplasm.

## Results

### Genome-wide structural variation

A CGH tiling microarray with 1.4 million features was used to estimate the genomic locations and sizes of SV events in the genomes of three classes of germplasm. The first class consisted of five transgenic plants each derived from a unique *Agrobacterium*-based transformation event. Each transgenic plant contained a different transgene (Table 1), and each event was specified by a unique Whole Plant Transformation (WPT) identifier. A range of different transgene types were represented among the five plants, including a green fluorescence protein (GFP) transgene, an RNAi hairpin, a zinc-finger nuclease (ZFN), a transcription activator-like effector nuclease (TALEN), and an mPing-Pong transposon. Genotyping was done on the T_1_ generation. Genome-wide CGH screens for deletions and duplications revealed single, unique SV in four of the five genotypes. These consisted of three deletions and one duplication (Table 1). The plant WPT_312-5-126 (ZFN transgene) did not exhibit any SV.

**Table 1:** Results from CGH, breakpoint sequencing, TAIL-PCR, and resequencing of transgenic plants

Transgenic Genotype	Construct	Data Types	Background	CGH-detected SV	T-DNA Location^a^	T-DNA Direction	SV adjacent to T-DNA	No. Transgenes
WPT_384-1-1	TALEN	CGH, TAIL-PCR	‘Bert-MN-01’	23,406 bp; Gm01 deletion	Gm07:35,729,562	+	Untested	Likely 1
WPT_389-2-2	mPing-Pong Transposon	CGH, NGS, TAIL-PCR, Southern Blot	‘Bert-MN-01’	125,228 bp; Gm11 deletion	Gm13:35,614,273	+	1,533 bp deletion + 37 bp deletion	1
WPT_301-3-13	GFP + RNAi Hairpin	CGH, TAIL-PCR, Southern Blot	‘Wm82-ISU-01’	6,869 bp; Gm13 duplication	Gm04:2,695,263	-	Untested	1
WPT_391-1-6	Magnesium Chelatase RNAi Hairpin	CGH, NGS	‘Bert-MN-01’	7,854 bp; Gm19 deletion	Gm05:38,834,281	+	~1,200 bp deletion	1
WPT_312-5-126	Zinc Finger Nuclease	CGH, Southern Blot	‘Bert-MN-01’	None	Untested	NA	Untested	1

^a^Genome coordinates adjacent to left border according to the soybean genome assembly version 1.0 (Wm82.a1.v1.1)

The second class, sampling FN-induced variation, consisted of a sub-set of plants from a larger mutant population developed in the genotype ‘M92-220’ [1]. This subset included ten plants with an associated mutant phenotype (Additional file 1: Table S1) and 35 plants that exhibited no obvious mutant phenotypes, and were thus referred to as “no-phenotype”. The final class, representing inter-cultivar variation, came from a previous study of genic SV [29], and consisted of 41 parental lines from a soybean Nested Association Mapping (SoyNAM) population (www.Soybase.org/SoyNAM).

All three datasets (transgenic, FN, and inter-cultivar) were designed to detect SV in each individual genotype as compared to an appropriate reference (Additional file 1: Table S2). The transgenic plants were compared to the transformation parent line (‘Bert’ [30] for four of the plants and ‘Williams 82’ for one plant; see Additional file 1: Table S2), the FN plants compared to the mutagenesis parent line (‘M92-220’), and the SoyNAM parents were compared to the reference genotype ‘Williams 82’. The Methods section includes analysis details and information on how extant heterogeneity within the background cultivars was addressed.

**Fig. 1:**
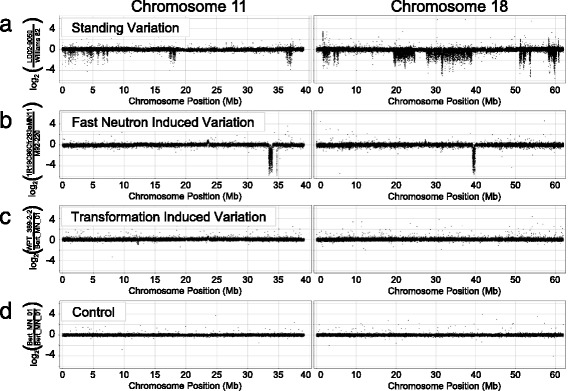
Visual comparison of CGH data for individuals from the three germplasm classes and control. Each *black dot* represents a single probe and its log_2_ ratio score. Data are shown from chromosome 11 on the left and chromosome 18 on the right for all samples. **a** The standing inter-cultivar variation between lines LD02-9050 and ‘Williams 82’ is shown. **b** Fast neutron (No-phenotype) plant 1R19C96Cfr293aMN11 is compared to the FN parent line ‘M92-220’. **c** Transgenic plant WPT_389-2-2 is compared to the parent line ‘Bert’; it shows relatively little noise and one true SV on chromosome 11. **d** The control CGH compared ‘Bert-MN-01’ to itself

As shown in Fig. 1, CGH results varied by chromosome and by class. In this figure each black dot represents a single probe’s log_2_ ratio score. Clusters of dots above or below zero are putative duplications or deletions, respectively. Inter-cultivar variation, shown as the comparison of SoyNAM parent LD02-9050 to ‘Williams 82’ (Fig. 1a), occurs frequently and on nearly every chromosome. The amount of inter-cultivar variation is strikingly high when compared to a FN or transgenic plant (Fig. 1b and c, respectively). The SV observed in FN or transformed plants was easier to detect, but occurred much less frequently.

**Fig. 2:**
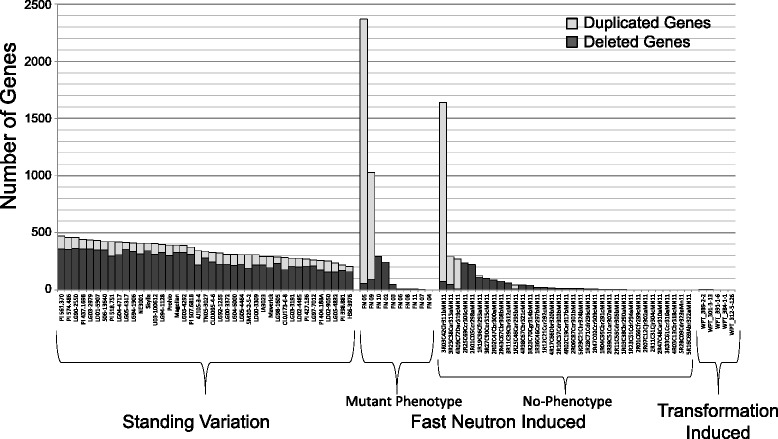
Distribution of genic SV from the three germplasm classes. Genic SV are found in individuals as standing variation in diverse cultivars (41 SoyNAM parents), induced by fast neutron mutagenesis (10 FN plants with a mutant phenotype and 35 FN plants with no obvious mutant phenotypes), or induced by the transformation process (five plants with unique constructs). Each column in the graph is a single genotype. *Light gray bars* represent “Duplicated Genes,” those overlapping putatively duplicated regions. *Dark gray bars* represent “Deleted Genes,” those overlapping putatively deleted regions

**Fig. 3:**
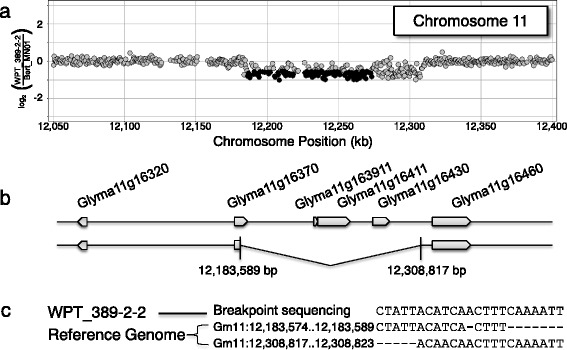
A novel deletion on chromosome 11 in transgenic plant WPT_389-2-2. **a** A plot of CGH data for the transgenic plant versus ‘Bert’ is shown, zoomed in on the chromosome 11 deletion seen in Fig. 1c. Probes are plotted as *dots* corresponding to the log_2_ ratio from the CGH array. *Dark gray dots* represent probes within significant SV segments that exceed the empirical threshold. Even with the extremely low detection threshold, part of this deletion could not be verified via CGH alone, necessitating visual inspection and sequencing of the deletion breakpoint. **b** Graphical interpretation of the hemizigous deletion found in WPT_389-2-2 is shown. **c** Sequence data from the breakpoint junction shows moderate homology on either end of the breakpoint

**Fig. 4:**
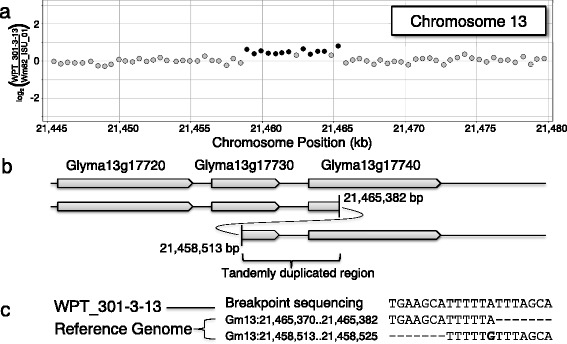
A novel duplication on chromosome 13 in transgenic plant WPT_301-3-13. **a** A plot of CGH data for the transgenic plant versus ‘Williams 82’ is shown, zoomed in on the chromosome 13 duplication. Probes are plotted as *dots* corresponding to the log_2_ ratio from the CGH array. *Dark gray dots* represent probes within significant SV segments that exceed the empirical threshold. **b** A graphical interpretation of the heterozygous duplication found in WPT_301-3-13, which includes a portion of Glyma13g17730 and a portion of Glyma13g17740, is shown. **c** The sequence data from the breakpoint junction shows five base pairs of homology on either end of the breakpoint

The number of genes putatively deleted or duplicated varied widely among the classes. Among the inter-cultivar comparisons, the total number of genes overlapping with duplications ranged from 45 to 124 per pairwise cultivar comparison, while the number of genes overlapping with deletions varied from 156 to 362 per comparison (Fig. 2). The FN class had a lower median genic SV per plant (Table 2) but was highly variable, as the number of genes overlapping with duplications ranged from 0 to 2,312 per plant, and the number of genes overlapping with deletions ranged from 0 to 290 per plant. The average size of the SV in the FN plants was over 500,000 bp, which is inflated by a small number of exceptionally large SV. Nevertheless, this value is substantially larger than those observed in the inter-cultivar class, where the average was less than 15,000 bp (Table 2). Of the four SV events in the transgenic plants, only two affected gene space. This included one deletion in plant WPT_389-2-2 that affected four genes on chromosome 11 (Fig. 3) and one duplication in plant WPT_301-3-13 that encompassed two genes on chromosome 13 (Fig. 4). Overall, the average number of genes affected by CGH-detectable SV in transgenic plants was estimated to be one order of magnitude less than that induced by FNs and two orders less than that observed among soybean varieties.

**Table 2:** Summary of SV frequency in Inter-cultivar, Fast Neutron (FN), and Transgenic genotypic classes

		Inter-Cultivar	FN Mutant Phenotype	FN No-Phenotype	Transgenic
Unique Up CNV Genes	Total genes in class	223	3253	2118	2
	Maximum among genotypes	124	2312	1568	2
	Median among genotypes	83	0	0	0
	Minimum among genotypes	45	0	0	0
Unique Down CNV Genes (homozygous or heterozygous deletions)	Total genes in class	1126	987	1231	4
	Maximum among genotypes	362	290	236	4
	Median among genotypes	244	48	12	0
	Minimum among genotypes	156	0	0	0
Up SV (homozygous duplications)	Total genic segments in class	117	11	9	1
	Mean Size	13,580 bp	4,671,937 bp	2,447,335 bp	6,434 bp
	Median Size	3,182 bp	2,802,275 bp	747,592 bp	6,434 bp
Down SV (homozygous or heterozygous deletion)	Total genic segments in class	547	23	49	1
	Mean Size	14,958 bp	1,276,033 bp	515,051 bp	125,228 bp
	Median Size	2,775 bp	110,656 bp	131,036 bp	125,228 bp

### Validation of SV in the transgenic plants

The four incidences of SV detected with CGH in the transgenic plants were confirmed using PCR. Two SV events overlapped with genes, including a 125,228 bp deletion on chromosome 11 in WPT_389-2-2 (Fig. 3) and a 6,869 bp duplication on chromosome 13 in WPT_301-3-13 (Fig. 4). The two non-genic deletions included 23,406 bp on chromosome 1 in WPT_384-1-1 (Additional file 2: Figure S1) and 7,854 bp on chromosome 19 in WPT_391-1-6 (Additional file 2: Figure S2). Sequence data from all four SV junctions showed evidence of microhomology-mediated DNA repair (Figs. 3c and 4c, and Additional file 2: Figure S1c and S2d).

Screening a subset of these SV by PCR confirmed they were not intra-cultivar variation in the ‘Bert’ or ‘Williams 82’ backgrounds, as is known to exist at some loci [31] (Additional file 2: Figure S3), or derived from contamination or outcrossing from other lines (Additional file 2: Figure S4). The deletions on chromosome 1 and chromosome 11 were stably inherited in T_1_ siblings and T_2_ offspring (Additional file 2: Figure S1 and S5), indicating these events were both present in their respective T_0_ generations. The deletion on chromosome 19 was homozygous and therefore present in the T_0_ generation assuming SV is induced on a single chromosome and then becomes a homozygous deletion through genetic segregation. These data indicate these SV were derived *de novo*. The duplication on chromosome 13, however, is not found in any individual other than the T_1_ transgenic genotype, WPT_301-3-13. The offspring (T_1:2_), siblings (T_1_), and parent (T_0_) of this individual were all tested and showed no evidence of the duplication on chromosome 13 (Additional file 2: Figure S6). This evidence suggests the duplication arose in a post transformation generation and may not be directly attributable to the transformation process.

### Transgene insertion sites

**Fig. 5:**
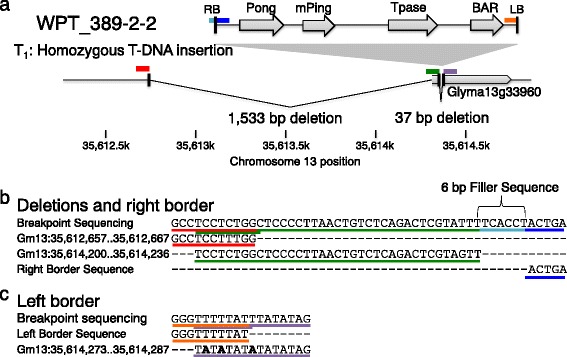
Transgene insertion locus and induced homozygous deletions in transgenic plant WPT_389-2-2. **a** A graphical interpretation of the transgene orientation and induced deletions at this locus is shown. The transgene insertion on chromosome 13 contains four primary elements between the left and right borders: Pong, mPing, Tpase, and BAR. *Colored lines* correspond to the breakpoint sequence results. **b** Results of breakpoint sequencing show a 1,533 bp deletion adjacent to the T-DNA right border (*dark blue*). The deletion results in a unique junction connecting two genomic segments (*red* and *green*) immediately adjacent to a 6 bp track of filler sequence (*light blue*), and then the T-DNA right border (*dark blue*). **c** A 37 bp deletion is found at the left border-genome junction (*orange* and *purple*, respectively). Microhomology occurs across the large deletion and between the left border and the genome

Transgenic plants were analyzed for number of transgene insertions and location of transgene(s). Southern blots of siblings or parents of WPT_301-3-13, WPT_312-5-126, and WPT_389-2-2 each showed evidence for single locus integration (Additional file 2: Figure S7). Thermal Asymmetric Interlaced PCR (TAIL-PCR) mapped the single insertion sites in WPT_389-2-2, WPT_384-1-1, and WPT_301-3-13. Resequencing data were also used to localize the T-DNA insertion site in WPT_389-2-2 and WPT_391-1-6. Transgene results are summarized in Table 1. Transgenes were all found to occur on different chromosomes than the aforementioned SV (Table 1). Transgene insertion and repair was observed to coincide with microhomology between the genome and the left border (Fig. 5 and Additional file 2: Figure S8).

According to the whole genome resequencing data from two transgenic plants, transgene insertions in WPT_389-2-2 and WPT_391-1-6 both induced adjacent deletions too small for CGH detection (the other three transgenic plants were not analyzed by whole genome resequencing). The deletion induced by the transgene insertion in WPT_391-1-6 was ~1,200 bp and occurred adjacent to the transgene (Additional file 2: Figure S9).

The transgene locus from WPT_389-2-2 was more complex. As outlined in Fig. 5a, the transgene (an mPing-Pong transposon construct) induced two deletions and a 6-bp insertion of filler sequence in the T-DNA integration process. This transgene integration and associated mutations occurred in the promoter region and 5′UTR of Glyma13g33960. The WPT_389-2-2 T-DNA and adjacent mutations were homozygous in this T_1_ plant. The resequencing data aligned to the transgene found nine read-pairs that spanned the mPing-Pong portion of the construct (Additional file 2: Figure S10a) suggesting one of the homologous chromosomes has a transgene where this mPing-Pong portion was deleted or jumped out (Additional file 2: Figure S10b), as has been demonstrated with this element [32]. Had this transposon reintegrated in the genome, the methodology used for transgene mapping should have detected it.

### Genome-wide single nucleotide substitutions

Resequencing data were used to assess the frequency of nucleotide substitutions within the inter-cultivar, FN, and transgenic classes. Based on earlier studies, it has been established that pairwise comparisons of soybean cultivars typically identify over one-million single base substitutions [33, 34]. We tested our substitution identification pipeline by resequencing cultivars ‘Archer’ and ‘Noir 1’. These data corroborated earlier studies, as ‘Archer’ and ‘Noir 1’ respectively exhibited 1,110,325 and 1,904,061 homozygous substitutions compared to the soybean reference genome ‘Williams 82’.

**Fig. 6:**
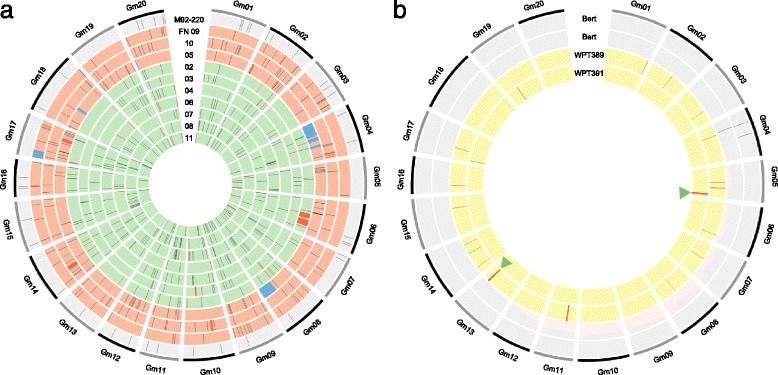
Genome wide view of induced variation detected through CGH and resequencing. The genomic locations of nucleotide substitutions (*black bars*), large duplications (*blue bars*), and large deletions (*bright red bars*) are shown for ten fast neutron plants (**a**) and two transgenic plants (**b**). Regions were filtered for background line heterogeneity such that only variants unique to one individual are shown. **a** Fast neutron plants, including the parent ‘M92-220’ (*outer ring*) and FN02-FN11 (*inner rings*) are shown. Background is shaded according to fast neutron irradiation dosage: *gray* is the non-irradiated parent ‘M92-220’, *light red* is 32 Gy (FN 09, 05 and 10), and *green* is 16 Gy (FN 02, 03, 04, 06, 07, 08, and 11). **b** Unique genetic variation in two different sequenced ‘Bert’ parent individuals (*gray background*), and transgenic plants WPT_391-1-6 and WPT_389-2-2 (*yellow backgrounds*) is shown. Transgene insertion sites are noted by *green arrows* and *bars*

Resequencing data were then used to assess the frequency of nucleotide substitutions in the previously sequenced ten mutant phenotype FN plants and the FN parent ‘M92-220’ [1] (Additional file 1: Table S1). Substitutions were detected and filtered so only those homozygous and novel to one plant were included. This filtering method was based on previous mutation accumulation studies [8, 35, 36]. The FN mutagenized plants had on the order of tens of unique homozygous substitutions per individual (Additional file 1: Table S3), with the highest individual exhibiting 73 substitutions. However, most of these substitutions may be attributed to spontaneous processes [36] rather than the FN treatment, as the nonmutagenized ‘M92-220’ control also exhibited 41 unique substitutions relative to the ten FN plants. As shown in Fig. 6a, substitutions in the FN plants were distributed across many more chromosomes than SV.

The two resequenced transgenic plants were also analyzed for homozygous and novel substitutions, along with two non-transgenic ‘Bert’ control plants (Additional file 1: Table S3). The number of novel homozygous base-pair substitutions per individual were as follows: two in plant WPT_391-1-6, eighteen in plant WPT_389-2-2, one in the first ‘Bert’ control plant, and two in the second ‘Bert’ control plant. The location of the substitutions in the transgenic plants appeared unrelated to the location of the transgene insertions or the induced SV (Fig. 6b) and did not occur in coding regions (Additional file 1: Table S3).

## Discussion

In this study, we observed the rates of SV and single nucleotide substitutions in transgenic and FN plants and explored the genetic nature of the unintended consequences of these breeding practices. The primary safety concern relating to these genomic changes is that novel genetic variants might disrupt genes or pathways leading to an unforeseen harmful byproduct [3]. Therefore, we focused our comparisons specifically on the protein-encoding gene space, with less emphasis on intergenic space and heterochromatin. Furthermore, we focused on the number of genes affected by new mutations rather than on the risk associated with a specific mutation or disruption of a specific gene. While the latter is of critical importance, it is impossible to estimate the specific effects of mutating each of the over 40,000 soybean genes. Therefore, for this discussion, differences in the number of total genes disrupted serves as a proxy for the amount of risk associated with each of these tools for genetic variation.

The SV observed in the inter-cultivar comparison was widespread throughout the genome, including many events that were repeatedly found in multiple lines and several events that encompassed only a single gene. This diversity has presumably accumulated through ongoing spontaneous mutation over numerous generations. Each of the genetic variants seen in this class would not be perceived to pose a new risk to consumers, as they likely already exist in the current marketplace. Furthermore, the genetic variation currently segregating in these lines represents only a subset of the total genetic diversity found in *Glycine max* or the wild progenitor *Glycine soja* [33, 34]. Genetic variation arising spontaneously, or introgressed from diverse lines into elite cultivars, is a process by which even cultivars developed through traditional breeding methodology unintentionally introduce novel variants to the marketplace.

The SV observed in the FN plants contrasts with the patterns of SV in the inter-cultivar class. SV induced through FN mutagenesis are oftentimes large and highly variable from plant to plant in terms of the number of genes affected. The large sizes of some of the SV observed in the no-phenotype FN plants were unexpected, as multigene deletions and duplications would be expected to cause noticeable phenotypic changes.

The transgenic class had so few SV that it is difficult to compare with the other classes. The events observed through CGH are moderate in size, impacting a combined total of only six genes among the five plants. While this likely represents a single generation increase in the SV mutation rate compared to the spontaneous SV mutation rate in soybeans, the total amount of genetic disturbance is substantially less than that observed in the standing soybean collection or the FN-induced plants. Working under the aforementioned assumption that each gene deleted or duplicated may pose a safety risk, one would conclude that the transgenic plants in this study are of lower risk than the vast majority of the FN plants analyzed, in terms of background genome disruption. Furthermore, while some induced variation may occur at the transgene locus, extensive backcrossing to introgress transgenes into elite backgrounds (which is the current common practice in many crop species) makes any new SV event(s) unlinked to the transgene inconsequential to the final cultivar.

While these transformation-induced events seem inconsequential when compared to those induced through FNs or found as standing variation, the novel SV identified in these plants exhibited several interesting properties. The discovery of locally induced deletions, the addition of filler sequence, and microhomology between the left border and the insertion site, corroborate previous patterns of T-DNA insertion in Arabidopsis [20]. The ~1 kb deletions found at transgene insertion sites in both of the resequenced soybean plants are substantially larger than the deletions previously found in Arabidopsis, but the sampling of only two plants is not sufficient to infer a general pattern. Short sequence homology was observed at the T-DNA insertion sites and also at the breakpoints of the four SV observed at non-transgene loci in these plants. These results imply that the microhomology-mediated end joining pathway [37] may be frequently involved in the DNA repair of these events.

The use of FN mutagenesis or tissue culture/transformation has been previously reported to result in a single generation increase in single nucleotide substitutions [8–11, 35]. A single nucleotide substitution disrupting a coding or regulatory region could similarly have an assumed safety risk associated with a novel byproduct. The FN and transgenic plants in this study accumulated a similar number of unique homozygous substitutions compared to a subset of previously published results. For example, a FN mutagenesis study in Arabidopsis detected between 5 and 18 novel homozygous substitutions per M_3_ plant [35] and a similar study of Arabidopsis tissue culture reported between 9 and 65 novel homozygous substitutions per R_1_ (the generation following tissue culture regeneration; analogous to T_1_) plant [8]. In rice, a FN mutagenesis study observed between 28 and 78 mutations per line in an M_3_ population, with the majority of mutations being single base substitutions [38], and a tissue culture study found no considerable difference in the number of variants in transgenic compared to control (wild type) plants [12]. In the present study, the number of unique homozygous substitutions observed in our control plants was similar to the number in the FN or transgenic plants, respectively. This implies that most of the identified substitutions were likely due to spontaneous mutation rather than a treatment effect of mutagenesis or transformation. In terms of single nucleotide substitutions, this result indicates that there is minimal difference in the safety risks associated with the three germplasm classes. This result stands in contrast to some of the previous studies of tissue culture in rice, where the authors concluded that there was a significantly higher number of induced homozygous substitutions and associated mutation rates [10, 11, 13]. A number of confounding factors might affect these incongruities, including differences in the species examined, SNP calling methods and thresholds, adjustments for intra-cultivar heterogeneity, FN dosage or tissue culture conditions and timeline, the inclusion of a control plant, and the number of plants sampled.

Based on data from the present study, it appears the use of FN mutagenesis can produce profound new SV events and may slightly increase the number of single nucleotide substitutions. Tissue culture/transformation methodologies can also produce new SV and possibly increase the nucleotide substitution rate. However, the number of SV and single nucleotide polymorphisms existing as standing variation in soybean cultivars dwarfs the induced variation observed in both FN and transformed plants. While these findings are noteworthy, it is unclear how broadly they can be applied. All of the transgenic plants in this study were obtained from *Agrobacterium*-mediated transformation; further work would test other transformation techniques such as biolistic-based methods. Similarly, FN irradiation was the only mutagenesis system tested; other mutagens (EMS, ENU, X-rays, etc.) would likely induce different mutational profiles. Furthermore, a deeper sampling of mutated and transformed plants, perhaps among different plant species, would be required to generalize the SV and nucleotide trends observed. Detailed sequence analysis of specific transgene loci did identify a small number of intermediate-sized deletions adjacent to transgenes, but there was no systematic attempt to detect intermediate-sized (1–2,000 bp) deletions/duplications genome-wide. Additional variants have also been reported to exist in FN [1, 38] and transgenic plants [12, 17, 39–41] but were not assessed within this dataset, including insertions, inversions and translocations, as well as epigenetic or transcriptional perturbations. Lastly, soybean is a palaeopolyploid species. It is likely that a true polyploid (or true diploid) species may exhibit differential tolerance or lack of tolerance to the type of genetic perturbations associated with these technologies.

## Conclusions

The total findings of this study help to inform the discussion currently surrounding the unintended consequences of genetic transformation in crop improvement [4, 42]. First, the frequency of induced SV events appears to be low, particularly in comparison to the frequency of those induced by FNs. Additionally, these rare SV events are likely indistinguishable from other spontaneously occurring SV or those already present in the existing germplasm. As demonstrated by the genetic variability in the no-phenotype FN plants, SV generated *de novo* are not necessarily associated with novel or noticeable phenotypic traits, even when these SV events are large. Therefore, the speculated risk of unintended genetic consequences in tissue culture/transformation may only merit as much consideration as given to variation arising spontaneously, through traditional breeding practices, or other genetic variation induction methods.

## Methods

### Plant materials and genetic transformation

The plant materials comprising the inter-cultivar and FN classes included in this study have been previously described [1, 29]. Briefly, the inter-cultivar group consists of 41 soybean accessions used as parents in developing the SoyNAM population. The FN population was developed in the background of the variety ‘M92-220’ [43] derived from the 2006 Crop Improvement Association seed stock of variety ‘MN1302’ [44]. Two types of FN plants were studied, including ten with detectable mutant phenotypes and 35 with no detectable phenotype. All FN plants were descendants of unique M_1_ individuals that were treated with either 4, 16, or 32 Gy of FN radiation [1].

Genetic transformation using *Agrobacterium rhizogenes* followed published methods [45, 46]. Each plant was confirmed to be transgenic based on PCR analysis and survival on selective (herbicide-treated) medium. The five T_1_ soybean individuals were from unique transformation events. The constructs for these transformations included a zinc finger nuclease [47], transcription activator-like effector nuclease, GFP and RNAi hairpin, mPing-Pong transposon [32], and a magnesium chelatase [48] RNAi hairpin. These transformations were in a ‘Bert’ cultivar [30] background (subline’Bert-MN-01’) or ‘Williams 82’ (subline ‘Wm82-ISU-01’) [31, 49]. The ‘Bert-MN-01’ subline (referred to as ‘Bert’ throughout this study) was derived from a single ‘Bert’ individual to reduce heterogeneity between transformed plants. The ‘Wm82-ISU-01’ subline (referred to as ‘Williams 82’ throughout this study) was derived from a single ‘Williams 82’ individual and is the nearest known match to the soybean reference genome assembly version 1.0 (Wm82.a1.v1.1) [31, 50].

### Comparative genome hybridization

The CGH data for all comparisons used in this study have been deposited in the National Center for Biotechnology Information Gene Expression Omnibus (http://www.ncbi.nlm.nih.gov/geo). The data for the inter-cultivar, FN, and transgenic plant comparisons can be found as accession numbers GSE56351, GSE58172, and GSE73596, respectively.

As with previous CGH analyses [1, 29], the DEVA software algorithm SegMt was used to generate raw data and identify segments in the transgenic plants. DNA samples from transgenic plants were labeled with Cy3 and the appropriate reference individual (‘Bert’ or ‘Williams 82’) was labeled with Cy5. Program parameters were: minimum segment difference = 0.1, minimum segment length (number of probes) = 2, acceptance percentile = 0.99, number of permutations = 10. Spatial correction and qspline normalization were applied. The resulting segments were processed based on their log_2_ ratio mean. Segments that exceeded the upper threshold were considered “UpCNV”. Segments that were less than the lower threshold were considered “DownCNV”. The upper threshold of 0.3484 and lower threshold of −0.5257 were based on empirical data from hemizygous deletions and duplications in eight previously characterized FN plants (Additional file 1: Table S4) [1]. A custom Perl script calculated the number of genes overlapping these significant segments. Minimum segment length was adjusted to three probes to account for noise seen in control arrays. Structural variants in the transgenic plants were further investigated through visual inspection, to identify any obvious SVs that were not detected by the threshold based pipeline.

SV attributable to intra-cultivar heterogeneity were removed, as has been done in the previous studies [1, 29]. Intra-cultivar heterogeneity was seen as significant segments of the exact same location occurring in multiple plants. By overlaying the raw CGH data of the four transgenic plants in the ‘Bert’ background, heterogeneous SV in the ‘Bert’ cultivar were removed. A similar method was used to filter out heterogeneity in the transformed ‘Williams 82’ background. The comparison array in this case was ‘Williams’ (the backcross parent in ‘Williams 82’ [49]) also hybridized to ‘Williams 82’. Any identical SV event discovered in both ‘Williams’ and transformed ‘Williams 82’ was considered heterogeneity and removed.

The CGH platform, methods, and filtering steps of the inter-cultivar and FN data have been previously described [1, 29]. The SV detected in the inter-cultivar variation study were all cross validated with resequencing data and conservative thresholds. For all CGH arrays, test genotypes were labeled with Cy3 and the appropriate reference individual was labeled with Cy5 in all hybridizations (Additional file 1: Table S2).

Visual displays of the CGH data were created using Spotfire DecisionSite software. Additional file 1: Table S5 provides a list of soybean plants chosen for analysis, corresponding publication, and hybridization reference. Our previous study [29] of inter-cultivar variation assessed CNV on a gene-by-gene cross-validated basis across all 41 SoyNAM genotypes, concluding that SV affected 1528 genes. We conservatively converted this to SV genes per genotype using the CGH thresholds from the study and probe-based log_2_ ratio score for each of the 1528 genes. FN data came from the “no-phenotype” class of 35 plants as described above, and ten “mutant phenotype” lines described in Additional file 1: Table S1 [1]. Only SV overlapping with genes were included in segment size summaries in all three genotypic classes.

### Confirming novel SV

PCR was used to confirm structural variants found with CGH in the transgenic plants. PCR and Sanger sequencing across breakpoints was used to confirm the four CGH observed events. Confirmed events and internal primers were used to genotype the structural variants in additional plants. Primer sequences are provided in Additional file 1: Table S6. In three of these lineages, siblings and offspring of the transgenic plants were genotyped to test if the SV were heritable. The events were confirmed not to be intra-cultivar heterogeneity by PCR-genotyping 47 untransformed individuals (either in the corresponding ‘Bert’ or ‘Williams 82’ background) at these three loci. Furthermore, the SoyNAM parents as well as cultivars ‘Archer’, ‘Minsoy’, and ‘Noir1’ were also PCR-genotyped with the breakpoint and internal primers to test for novelty of the SV events.

### Analyzing transgene insertion sites

Transgene integrations were analyzed using TAIL-PCR [51], Southern blot, and resequencing data. Southern blots used a BAR gene probe to detect the number of T-DNA insertions in the plants tested. TAIL-PCR was used to detect T-DNA locations in WPT_384-1-1, WPT_389-2-2 and WPT_301-3-13. Transgene insertion sites and counts were also determined by resequencing according to steps one through six outlined by Srivastava et al. [52]. Briefly, raw paired-end reads were aligned using Bowtie2 to the transgene sequence between the left and right border and the orphaned mapped reads were then aligned to the host soybean genome. The resulting putative transgene integration locations were filtered on prior knowledge of homology between components of the transgene (i.e. GmUbi promoter, RNAi hairpin targets, and their paralogs) and the genome. The location of the mapped orphaned reads, read depth coverage, and paired-end read spacing were further used to detect SV induced locally to transgene insertions. Integrated Genome Viewer (IGV) version 2.3.52 was used to visualize alignment results [53].

### Sequence handling, alignment, and calling of nucleotide substitutions

The sequence read data from the ten “mutant phenotype” fast neutron plants analyzed in this study, along with the parent line of the population (cv. ‘M92-220’), are deposited in the Sequence Read Archive (http://www.ncbi.nlm.nih.gov/sra/) under accession number SRP036841. The sequence read data from the two transgenic plants, along with two individuals of the parent line (cv. ‘Bert’), and the cultivars ‘Archer’ and ‘Noir 1’ are deposited in the Sequence Read Archive under accession number SRP063738.

To determine the relative rates of base substitution due to FN mutagenesis, we used resequencing data from the aforementioned ten FN plants that had associated mutant phenotypes as reported in [1] (see Additional file 1: Table S1). We sequenced two transgenic plants and two controls to estimate the base substitution rate and localize T-DNA insertion sites. See Additional file 2: Figure S11 for the transgenic resequencing data analysis pipeline. All individuals were sequenced with Illumina 100 bp paired end reads.

FastQC version 0.11.2 was used on initial read data (and after any modifications to sequence data) to ensure that tools were used properly and the data was of acceptable quality for downstream applications [54]. Forward and reverse reads were treated separately, and then resynchronized for alignment using resync.pl (Riss util version 1.0, http://msi-riss.readthedocs.org/en/latest/software/riss_util.html). Cutadapt version 1.6 was used to remove adapter sequences using –b to specify both adapter sequences (GATCGGAAGAGCACACGTCTGAACTCCAGTCAC-NNNNNN-ATCTCGT-ATGCCGTCTTCTGCTTG, AATGATACGGCGACCACCGAGATCTACACTCTTTCCC-TACACGACGCTCTTCC-GATCT) where NNNNNN specifies the unique 6 bp sequence attached to samples when multiplexing. Sequence artifacts (low-complexity reads) were removed using fastx artifacts filter (Fastx toolkit version 0.0.14). Read quality was further filtered using fastq quality trimmer in the fastxtoolkit. Bases with phred quality of less than 20 were removed, and reads that were shorter than 30 bp after trimming were discarded.

We chose to align reads to the reference with two different read mapping programs, BWA mem (v. 0.7.10) [55], and Bowtie2 (v. 2.2.4) [56]. BWA mem alignments allowed for more accurate single base substitution calls, and Bowtie2 produces alignments more suitable for confirming CGH-identified SV. For BWA mem, the mismatch penalty was set to 6 (−B 6), which allows for approximately seven high-quality mismatches per read. Bowtie2 alignments were produced with default parameters. In both cases, reads were mapped to the *Glycine max* assembly version 1.0 (Wm82.a1.v1.1) [50]. Read cleaning and post-alignment filtering resulted in a realized mean coverage of 35x for the FN mutagenized plants, and 20x for WPT_389-2-2, and 21x for WPT_391-1-6.

Genotype calls for all sites were generated with the UnifiedGenotyper in the Genome Analysis Tool Kit (GATK) version 3.3 [57]. Pairwise comparisons of soybean varieties typically identify over one-million single base substitutions [33, 34]. This BWA mem resequencing and SNP detection pathway identified 1,110,325 substitutions between genotype ‘Archer’ and the ‘Williams 82’ reference genome sequence, and 1,904,061 substitutions between genotype ‘Noir 1’ and ‘Williams 82’. These findings served as a control to demonstrate our analysis pipeline identified similar polymorphism counts as have been previously reported in soybean studies.

We then applied a set of filtering criteria to look at only substitutions that are private to a single individual (termed “unique” or “novel” throughout the paper) across the most confidently called portions of the genome. This excluded sites with less than five reads per sample, sites that were monomorphic for the reference base, sites with heterozygous or missing calls, and sites with a homozygous alternate base call in more than one individual. Applied together, these filtering criteria produced variant calls that were homozygous private differences from reference. The filtering criteria assumed *de novo* mutations at a single base position will only be observed once. A large section in FN plant 07 on Chromosome 12 between 10 and 23 Mb was found to contain a disproportionate number of substitutions. CGH results from other FN individuals [1], not included in this sample, suggest this region is heterogeneous in the ‘M92-220’ cultivar. We therefore excluded this region of 183 substitutions when analyzing FN plant 07. The observed transition:transversion ratios were too variable between individuals to compare to previously reported ratios in FN mutagenesis [35].

Circos plots [58] were generated using 2d tile data tracks, plotting unique substitutions detected, previously published FN-induced SV [1], detected transformation-induced SV, and T-DNA mapping results. Scripts to perform data handling and analysis are available at https://github.com/TomJKono/Unintended_Consequences.

### Availability of supporting data

The data sets supporting the results of this article are available in the National Center for Biotechnology Information Gene Expression Omnibus (http://www.ncbi.nlm.nih.gov/geo) and Sequence Read Archive (http://www.ncbi.nlm.nih.gov/sra/) repositories, GSE56351, GSE58172, GSE73596, SRP036841, and SRP063738.

## Additional files

Additional file 1: Table S1. Resequenced fast neutron genotypes, all from the forward screen family, Bolon et al. [1]. **Table S2.** Summary of data type, CGH design, and analysis method for Inter-cultivar, Fast Neutron, and Transgenic genotypic classes. **Table S3.** Summary of SNP frequencies in a subsample fast neutron and transgenic plants. **Table S4.** Genotypes and regions used to develop CGH log_2_ ratio empirical thresholds. **Table S5.** Genotypes examined by CGH. **Table S6.** Sequences of PCR primers used for genotyping. (DOCX 48 kb) Additional file 2: Figure S1. A novel deletion detected on chromosome 01 in transgenic plant WPT_384-1-1. **Figure S2.** A novel deletion on chromosome 19 in transgenic plant WPT_391-1-6. **Figure S3.** Test for intracultivar variation in the parental lines by genotyping 47 individuals taken from GRIN stocks of the varieties ‘Bert’ and ‘Williams 82’. **Figure S4.** Genotyping diverse lines including the 41 SoyNAM parents, cultivars ‘Archer’, ‘Minsoy’, and ‘Noir1’, ‘Bert-MN-01’, and ‘Wm82-ISU-01,’ for previous evidence of SV found in transformed plants. **Figure S5.** Novel deletion on chromosome 11 in transgenic plant WPT_389-2-2. **Figure S6.** Novel duplication on chromosome 13 in transgenic plant WPT_301-3-13. **Figure S7.** Southern blot analysis of *Hind*III digested genomic DNA. **Figure S8.** Microhomology of sequences at the T-DNA left border and the sites of genomic integration for three transgenic plants. **Figure S9.** Structure of the heterozygous transgene insertion on chromosome 05 in transgenic plant WPT_391-1-6. **Figure S10.** Transgene insertion on chromosome 13 in transgenic plant WPT_389-2-2. **Figure S11.** Pipeline for utilizing resequencing data in this study. (PPTX 3058 kb)

## Abbreviations

CGH: array-based comparative genomic hybridization
FN: fast neutron
GFP: green fluorescence protein
NGS: next-generation sequencing
SoyNAM: soybean nested association mapping
SV: structural variants
TAIL-PCR: thermal asymmetric interlaced PCR
TALEN: transcription activator-like effector nuclease
WPT: whole plant transformation
ZFN: zinc-finger nuclease
