# Supplementary material for: Genomic variation and DNA repair associated with soybean transgenesis: a comparison to cultivars and mutagenized plants
Source: BMC Biotechnol. 2016 May 12;16:41. doi: 10.1186/s12896-016-0271-z (PMC4866027; doi:10.1186/s12896-016-0271-z)
Supplement: Additional file 1: Table S1. — Resequenced fast neutron genotypes, all from the forward screen family, Bolon et al. [1]. Table S2. Summary of data type, CGH design, and analysis method for Inter-cultivar, Fast Neutron, and Transgenic genotypic classes. Table S3. Summary of SNP frequencies in a subsample fast neutron and transgenic plants. Table S4. Genotypes and regions used to develop CGH log2 ratio empirical thresholds. Table S5. Genotypes examined by CGH. Table S6. Sequences of PCR primers used for genotyping. (DOCX 48 kb) [file 12896_2016_271_MOESM1_ESM.docx]

**Additional file 1: Supplementary Tables**

Table S1. Phenotypes and structural variation information for the ten fast neutron lines included in this study that were determined to have a mutant phenotype (Bolon et al., 2014).

| Soybase ID | Coded Name (This study) | Mean Coverage (BWA) | Putative deleted genes | No. chromos. with deleted genes | Putative duplicated genes | No. chromos. with duplicated genes | Soybase Family Name | CGH ID | M2 Family Name | Rad. Dose | Gen. | Mutant phenotype |
| --- | --- | --- | --- | --- | --- | --- | --- | --- | --- | --- | --- | --- |
| M92-220.x1.04.WT | FN01 (M92-220 - Long) | 64 | - | - | - | - | - | - | - | - | - | - |
| FN0173217.03.09.01.M5 | FN02 | 37 | 243 | 2 | 0 | 0 | FN0173217 | R32C17P18C09 #1 rep2 | R32C17CSCW08YB | 16 Gy | M5 | High seed protein |
| FN0172932.09.08.01.M5 | FN03 | 26 | 48 | 2 | 0 | 0 | FN0172932 | R29C32P13i08 #1 rep2 | R29C32CSCW08YB | 16 Gy | M5 | High seed weight, low seed protein |
| FN0175143.05.06.01.M5 | FN04 | 36 | 0 | 0 | 0 | 0 | FN0175143 | R51C43P26e06 #1 rep2 | R51C43CSCW08YB | 16 Gy | M5 | High seed oil, high seed protein and oil |
| FN0171501.01.02.M4 | FN05 | 31 | 56 | 2 | 2312 | 3 | FN0171501 | R15C01P33a02 | R15C01DSCW08YB | 32 Gy | M4 | High seed protein |
| FN0131633.06.01.M4 | FN06 | 34 | 7 | 2 | 0 | 0 | FN0131633 | 3R16C33Cfr371aMN12 | 3R16C33CMN09NSFBV | 16 Gy | M4 | High seed oil, high seed protein and oil, high seed yield |
| FN0112228.06.02.01.M5 | FN07 | 34 | 2 | 1 | 0 | 0 | FN0112228 | 1R22C28Cfbr62aMN12 | 1R22C28CMN09NSFBV | 16 Gy | M5 | High seed oil, low seed protein, high seed yield, late maturity, short, bushy, and indeterminate |
| FN0112885.02.06.03.M5 | FN08 | 39 | 0 | 0 | 6 | 2 | FN0112885 | 1R28C85Cbfr55cMN12 | 1R28C85CMN09NSFBV | 16 Gy | M5 | High seed oil, low seed protein, high seed yield, late maturity, short, bushy, and indeterminate |
| FN0163764.04.01.M4 | FN09 | 22 | 92 | 3 | 934 | 2 | FN0163764 | 6R37C64Ddr229aMN12 rep2 | 6R37C64DMN09NSFBV | 32 Gy | M4 | Stunted, short internodes, short petiole, slightly lanceolate leaves, early maturity, determinate |
| FN0164160.03.02.01.01.M6 | FN10 | 32 | 290 | 3 | 1 | 1 | FN0164160 | 6R41C60Dcbar163aMN12 | 6R41C60DMN09NSFBV | 32 Gy | M6 | Seed composition mutant, small plant, slightly chlorotic, slightly rugose, slightly tawny pubescence |
| FN0175501.x2.02.01.M5 | FN11 | 30 | 6 | 2 | 0 | 0 | FN0175501 | GMGC2ba | R55C01CSCW08YB | 16 Gy | M5 | Short trichomes |

Table S2. Summary of data type, CGH design, and analysis method for the initial calling of structural genomic variation in the Inter-cultivar, Fast Neutron, and Transgenic genotypic classes.

|  | Inter-Cultivar | Fast Neutron | Transgenic |
| --- | --- | --- | --- |
| Original Experiment | Anderson et al., 2014 | Bolon et al., 2014 | Present Study |
| No. Genotypes Analyzed | 41 | 45 | 5 |
| Genotype Tested (Cy3) | SoyNAM Parent Accession | Mutant | Transformed Individual (T1) |
| Reference (Cy5) | ‘Wm82-ISU-01’ | ‘M92-220 – Long’ | ‘Bert-MN-01’ or ‘Wm82-ISU-01’ |
| Data Types | CGH & Whole Genome Sequence | CGH | CGH |
| Analysis Method | Cross validation, visual analysis | Array based thresholds, visual analysis | Empirical thresholds, visual analysis |
| Experiment designed to detect | Genes affected by SV | SV induced genome-wide | SV induced genome-wide |

Table S3. Summary of SNP frequencies in a subsample fast neutron and transgenic plants.

|  | FN01 M92-220 | FN02 | FN03 | FN04 | FN06 | FN07 | FN08 | FN11 | FN05 | FN09 | FN10 | Bert-1 | Bert-2 | WPT389-2-2 | WPT391-1-6 |
| --- | --- | --- | --- | --- | --- | --- | --- | --- | --- | --- | --- | --- | --- | --- | --- |
| Dosage | NA | 16 Gy | 16 Gy | 16 Gy | 16 Gy | 16 Gy | 16 Gy | 16 Gy | 32 Gy | 32 Gy | 32 Gy | - | - | - | - |
| Generation | - | M5 | M5 | M5 | M4 | M5 | M5 | M5 | M4 | M4 | M6 | - | - | T1 | T1 |
| Homozygous Substitutions | 41 | 45 | 42 | 41 | 49 | 58 | 62 | 44 | 76 | 50 | 73 | 2 | 1 | 18 | 2 |
| Genic |  |  |  |  |  |  |  |  |  |  |  |  |  |  |  |
| Coding | 2 | 1 | 4 | 2 | 1 | 5 | 16 | 4 | 3 | 1 | 6 | 0 | 0 | 0 | 0 |
| Non-Coding | 3 | 5 | 2 | 5 | 9 | 21 | 10 | 4 | 7 | 1 | 4 | 0 | 1 | 1 | 0 |
| Non-Genic | 36 | 39 | 36 | 34 | 39 | 32 | 36 | 36 | 66 | 48 | 63 | 2 | 0 | 17 | 2 |
| Ti:Tv Ratio | - | 2.4 | 1.7 | 1.9 | 1.2 | 3.0 | 0.8 | 1.2 | 1.3 | 1.9 | 1.4 | - | - | 1.5 | 0 |

Table S4. Genotypes and regions used to develop CGH log_2_ ratio empirical thresholds.

| Genotype | Segment Type | Chromosome | Average log_2_ Ratio | No. probes | Region Start | Region Stop | Region Size | Used as Universal Threshold |
| --- | --- | --- | --- | --- | --- | --- | --- | --- |
| 3R16C33Cfr371aMN12 | Hemizygous Deletion | GM16 | -0.525706452 | 899 | 8161171 | 8737551 | 576380 | Yes |
| 5R15C49Dcdr81aMN12 | Deletion | GM07 | -0.589188374 | 2116 | 28900343 | 30975759 | 2075416 |  |
| 6R41C60Dcbar163aMN12 | Deletion | GM04 | -0.731 | 2640 | 42480798 | 43845671 | 1364874 |  |
| R02C28-7-35-1 | Deletion | GM07 | -0.657297156 | 5662 | 24452904 | 31229508 | 6776604 |  |
| R07C12-6-41-1 | Deletion | GM15 | -0.7267 | 3332 | 43545233 | 46011969 | 2466737 |  |
| R32C17P18C09 #1 | Deletion | GM06 | -0.634165475 | 5593 | 22989683 | 29272095 | 6282412 |  |
| R32C17P18C09 #1 | Deletion | GM06 | -0.584217327 | 9067 | 31864518 | 39994964 | 8130446 |  |
| 6R41C60Dcbar163aMN12 | Duplication | GM04 | 0.4252 | 2955 | 43846110 | 45384387 | 1538278 |  |
| R02C28-7-35-1 | Duplication | GM15 | 0.376373176 | 74406 | 1 | 50938913 | 50938912 |  |
| R07C12-6-41-1 | Duplication | GM15 | 0.384310595 | 60774 | 1 | 42984752 | 42984751 |  |
| R07C12-6-41-1 | Duplication | GM15 | 0.42241886 | 8033 | 46567523 | 50938913 | 4371390 |  |
| R07C12-6-41-1 | Duplication | GM16 | 0.8113 | 3511 | 35311624 | 37131684 | 1820061 |  |
| R15C01P33a02 | Duplication | GM04 | 0.390922236 | 28090 | 81 | 16866782 | 16866701 |  |
| R15C01P33a02 | Duplication | GM04 | 0.3484 | 3272 | 19534674 | 23730015 | 4195342 | Yes |
| R15C01P33a02 | Duplication | GM04 | 0.3569 | 1885 | 29586547 | 31624484 | 2037938 |  |
| R15C01P33a02 | Duplication | GM08 | 0.356043871 | 8256 | 29455508 | 36860787 | 7405279 |  |
| R15C01P33a02 | Duplication | GM08 | 0.396 | 17935 | 36862182 | 46994705 | 10132524 |  |
| R15C01P33a02 | Duplication | GM18 | 0.3598 | 2284 | 24740911 | 27269292 | 2528382 |  |
| RP69dm4MNS12 | Duplication | GM03 | 0.507063898 | 3166 | 28675179 | 30920725 | 2245546 |  |

Table S5. Genotypes examined by CGH.

| Class | Genotype Tested | Hybridized to Genotype: | Publication | Radiation  Dose | Generation | GEO Series | GEO Accession |
| --- | --- | --- | --- | --- | --- | --- | --- |
| Transgenic | WPT0389-2-2_mPingline | ‘Bert-MN-01’ | This study | - | T1 | GSE73596 | GSM1898745 |
| Transgenic | WPT0391-1-6_MinnGold_hp | ‘Bert-MN-01’ | This study | - | T1 | GSE73596 | GSM1898746 |
| Transgenic | WPT0384-1-1_TALEN_Dcl2b | ‘Bert-MN-01’ | This study | - | T1 | GSE73596 | GSM1898744 |
| Transgenic | WPT_312_5_126_ZFN | ‘Bert-MN-01’ | This study | - | T1 | GSE73596 | GSM1898743 |
| Transgenic | WPT_301_3_13_GFP_RNAi Hairpin | ‘Wm82-ISU-01’ | This study | - | T1 | GSE73596 | GSM1898742 |
| Control | Bert-MN-01 | ‘Bert-MN-01’ | This study | - | - | GSE73596 | GSM1898747 |
| Control | Williams | ‘Wm82-ISU-01’ | This study | - | - | GSE73596 | GSM1898748 |
| NAM parent | TN05-3027 _NAM 02 | ‘Wm82-ISU-01’ | Anderson et al., 2014 | - | - | GSE56351 | GSM1359718 |
| NAM parent | 4J105-3-4_NAM 03 | ‘Wm82-ISU-01’ | Anderson et al., 2014 | - | - | GSE56351 | GSM1359719 |
| NAM parent | 5M20-2-5-2_NAM 04 | ‘Wm82-ISU-01’ | Anderson et al., 2014 | - | - | GSE56351 | GSM1359720 |
| NAM parent | CL0J095-4-6_NAM 05 | ‘Wm82-ISU-01’ | Anderson et al., 2014 | - | - | GSE56351 | GSM1359721 |
| NAM parent | CL0J173-6-8_NAM 06 | ‘Wm82-ISU-01’ | Anderson et al., 2014 | - | - | GSE56351 | GSM1359722 |
| NAM parent | HS6-3976 _NAM 08 | ‘Wm82-ISU-01’ | Anderson et al., 2014 | - | - | GSE56351 | GSM1359723 |
| NAM parent | Prohio_NAM 09 | ‘Wm82-ISU-01’ | Anderson et al., 2014 | - | - | GSE56351 | GSM1359724 |
| NAM parent | LD00-3309_NAM 10 | ‘Wm82-ISU-01’ | Anderson et al., 2014 | - | - | GSE56351 | GSM1359725 |
| NAM parent | LD01-5907 _NAM 11 | ‘Wm82-ISU-01’ | Anderson et al., 2014 | - | - | GSE56351 | GSM1359726 |
| NAM parent | LD02-4485_NAM 12 | ‘Wm82-ISU-01’ | Anderson et al., 2014 | - | - | GSE56351 | GSM1359727 |
| NAM parent | LD02-9050 _NAM 13 | ‘Wm82-ISU-01’ | Anderson et al., 2014 | - | - | GSE56351 | GSM1359728 |
| NAM parent | Magellan_NAM 14 | ‘Wm82-ISU-01’ | Anderson et al., 2014 | - | - | GSE56351 | GSM1359729 |
| NAM parent | Maverick_NAM 15 | ‘Wm82-ISU-01’ | Anderson et al., 2014 | - | - | GSE56351 | GSM1359730 |
| NAM parent | S06-13640 _NAM 17 | ‘Wm82-ISU-01’ | Anderson et al., 2014 | - | - | GSE56351 | GSM1359731 |
| NAM parent | NE3001_NAM 18 | ‘Wm82-ISU-01’ | Anderson et al., 2014 | - | - | GSE56351 | GSM1359732 |
| NAM parent | Skylla _NAM 22 | ‘Wm82-ISU-01’ | Anderson et al., 2014 | - | - | GSE56351 | GSM1359733 |
| NAM parent | U03-100612 _NAM 23 | ‘Wm82-ISU-01’ | Anderson et al., 2014 | - | - | GSE56351 | GSM1359734 |
| NAM parent | LG03-2979 _NAM 24 | ‘Wm82-ISU-01’ | Anderson et al., 2014 | - | - | GSE56351 | GSM1359735 |
| NAM parent | LG03-3191 _NAM 25 | ‘Wm82-ISU-01’ | Anderson et al., 2014 | - | - | GSE56351 | GSM1359736 |
| NAM parent | LG04-4717 _NAM 26 | ‘Wm82-ISU-01’ | Anderson et al., 2014 | - | - | GSE56351 | GSM1359737 |
| NAM parent | LG05-4292_NAM 27 | ‘Wm82-ISU-01’ | Anderson et al., 2014 | - | - | GSE56351 | GSM1359738 |
| NAM parent | LG05-4317_NAM 28 | ‘Wm82-ISU-01’ | Anderson et al., 2014 | - | - | GSE56351 | GSM1359739 |
| NAM parent | LG05-4464_NAM 29 | ‘Wm82-ISU-01’ | Anderson et al., 2014 | - | - | GSE56351 | GSM1359740 |
| NAM parent | LG05-4832_NAM 30 | ‘Wm82-ISU-01’ | Anderson et al., 2014 | - | - | GSE56351 | GSM1359741 |
| NAM parent | LG90-2550 _NAM 31 | ‘Wm82-ISU-01’ | Anderson et al., 2014 | - | - | GSE56351 | GSM1359742 |
| NAM parent | LG92-1255_NAM 32 | ‘Wm82-ISU-01’ | Anderson et al., 2014 | - | - | GSE56351 | GSM1359743 |
| NAM parent | LG94-1128 _NAM 33 | ‘Wm82-ISU-01’ | Anderson et al., 2014 | - | - | GSE56351 | GSM1359744 |
| NAM parent | LG94-1906_NAM 34 | ‘Wm82-ISU-01’ | Anderson et al., 2014 | - | - | GSE56351 | GSM1359745 |
| NAM parent | LG97-7012_NAM 36 | ‘Wm82-ISU-01’ | Anderson et al., 2014 | - | - | GSE56351 | GSM1359746 |
| NAM parent | LG98-1605 _NAM 37 | ‘Wm82-ISU-01’ | Anderson et al., 2014 | - | - | GSE56351 | GSM1359747 |
| NAM parent | LG00-3372 _NAM 38 | ‘Wm82-ISU-01’ | Anderson et al., 2014 | - | - | GSE56351 | GSM1359748 |
| NAM parent | LG04-6000 _NAM 39 | ‘Wm82-ISU-01’ | Anderson et al., 2014 | - | - | GSE56351 | GSM1359749 |
| NAM parent | PI 398.881_NAM 40 | ‘Wm82-ISU-01’ | Anderson et al., 2014 | - | - | GSE56351 | GSM1359750 |
| NAM parent | PI 427.136_NAM 41 | ‘Wm82-ISU-01’ | Anderson et al., 2014 | - | - | GSE56351 | GSM1359751 |
| NAM parent | PI 437.169B_NAM 42 | ‘Wm82-ISU-01’ | Anderson et al., 2014 | - | - | GSE56351 | GSM1359752 |
| NAM parent | PI 507.681B_NAM 46 | ‘Wm82-ISU-01’ | Anderson et al., 2014 | - | - | GSE56351 | GSM1359753 |
| NAM parent | PI 518.751_NAM 48 | ‘Wm82-ISU-01’ | Anderson et al., 2014 | - | - | GSE56351 | GSM1359754 |
| NAM parent | PI 561.370_NAM 50 | ‘Wm82-ISU-01’ | Anderson et al., 2014 | - | - | GSE56351 | GSM1359755 |
| NAM parent | PI 404.188A _NAM 54 | ‘Wm82-ISU-01’ | Anderson et al., 2014 | - | - | GSE56351 | GSM1359756 |
| NAM parent | PI 574.486_NAM 64 | ‘Wm82-ISU-01’ | Anderson et al., 2014 | - | - | GSE56351 | GSM1359757 |
| NAM parent | IA3023_NAM Universal Parent | ‘Wm82-ISU-01’ | Anderson et al., 2014 | - | - | GSE56351 | GSM1359758 |
| Fast Neutron  (No-Phenotype) | 1R03C38Cbr290aMN11 | ‘M92-220 – Long’ | Bolon et al., 2014 | 16 Gy | M4 | GSE58172 | GSM1402584 |
| Fast Neutron  (No-Phenotype) | 1R04C95Cbr291cMN11 | ‘M92-220 – Long’ | Bolon et al., 2014 | 16 Gy | M4 | GSE58172 | GSM1402585 |
| Fast Neutron  (No-Phenotype) | 1R12C21Ccr292cMN11 | ‘M92-220 – Long’ | Bolon et al., 2014 | 16 Gy | M4 | GSE58172 | GSM1402586 |
| Fast Neutron  (No-Phenotype) | 1R19C96Cfr293aMN11 | ‘M92-220 – Long’ | Bolon et al., 2014 | 16 Gy | M4 | GSE58172 | GSM1402587 |
| Fast Neutron  (No-Phenotype) | 1R23C51Cdr294aMN11 | ‘M92-220 – Long’ | Bolon et al., 2014 | 16 Gy | M4 | GSE58172 | GSM1402589 |
| Fast Neutron  (No-Phenotype) | 1R25C46Car295bMN11 | ‘M92-220 – Long’ | Bolon et al., 2014 | 16 Gy | M4 | GSE58172 | GSM1402590 |
| Fast Neutron  (No-Phenotype) | 1R28C71Cdr296cMN11 | ‘M92-220 – Long’ | Bolon et al., 2014 | 16 Gy | M4 | GSE58172 | GSM1402591 |
| Fast Neutron  (No-Phenotype) | 1R36C46Ccr297bMN11 | ‘M92-220 – Long’ | Bolon et al., 2014 | 16 Gy | M4 | GSE58172 | GSM1402593 |
| Fast Neutron  (No-Phenotype) | 2R01C05Ccr298aMN11 | ‘M92-220 – Long’ | Bolon et al., 2014 | 16 Gy | M4 | GSE58172 | GSM1402641 |
| Fast Neutron  (No-Phenotype) | 2R01C66Cfr299cMN11 | ‘M92-220 – Long’ | Bolon et al., 2014 | 16 Gy | M4 | GSE58172 | GSM1402642 |
| Fast Neutron  (No-Phenotype) | 2R02C47Cbr300aMN11 | ‘M92-220 – Long’ | Bolon et al., 2014 | 16 Gy | M4 | GSE58172 | GSM1402643 |
| Fast Neutron  (No-Phenotype) | 2R06C87Ccr301bMN11 | ‘M92-220 – Long’ | Bolon et al., 2014 | 16 Gy | M4 | GSE58172 | GSM1402644 |
| Fast Neutron  (No-Phenotype) | 2R07C12Cjr302aMN11 | ‘M92-220 – Long’ | Bolon et al., 2014 | 16 Gy | M4 | GSE58172 | GSM1402645 |
| Fast Neutron  (No-Phenotype) | 2R10C37Cdr303bMN11 | ‘M92-220 – Long’ | Bolon et al., 2014 | 16 Gy | M4 | GSE58172 | GSM1402646 |
| Fast Neutron  (No-Phenotype) | 2R11C31Cjr304cMN11 | ‘M92-220 – Long’ | Bolon et al., 2014 | 16 Gy | M4 | GSE58172 | GSM1402647 |
| Fast Neutron  (No-Phenotype) | 2R11C55Cdr305bMN11 | ‘M92-220 – Long’ | Bolon et al., 2014 | 16 Gy | M4 | GSE58172 | GSM1402648 |
| Fast Neutron  (No-Phenotype) | 2R25C69Ccr306cMN11 | ‘M92-220 – Long’ | Bolon et al., 2014 | 16 Gy | M4 | GSE58172 | GSM1402649 |
| Fast Neutron  (No-Phenotype) | 2R39C51Car307aMN11 | ‘M92-220 – Long’ | Bolon et al., 2014 | 16 Gy | M4 | GSE58172 | GSM1402655 |
| Fast Neutron  (No-Phenotype) | 2R43C67Cbr308bMN11 | ‘M92-220 – Long’ | Bolon et al., 2014 | 16 Gy | M4 | GSE58172 | GSM1402656 |
| Fast Neutron  (No-Phenotype) | 2R47C02Ccr309cMN11 | ‘M92-220 – Long’ | Bolon et al., 2014 | 16 Gy | M4 | GSE58172 | GSM1402658 |
| Fast Neutron  (No-Phenotype) | 2R47C48Car310aMN11 | ‘M92-220 – Long’ | Bolon et al., 2014 | 16 Gy | M4 | GSE58172 | GSM1402659 |
| Fast Neutron  (No-Phenotype) | 3R03C42Clr311bMN11 | ‘M92-220 – Long’ | Bolon et al., 2014 | 16 Gy | M4 | GSE58172 | GSM1402660 |
| Fast Neutron  (No-Phenotype) | 3R11C39Cbr312cMN11 | ‘M92-220 – Long’ | Bolon et al., 2014 | 16 Gy | M4 | GSE58172 | GSM1402661 |
| Fast Neutron  (No-Phenotype) | 3R23C38Car313aMN11 | ‘M92-220 – Long’ | Bolon et al., 2014 | 16 Gy | M4 | GSE58172 | GSM1402663 |
| Fast Neutron  (No-Phenotype) | 3R23C70Cgr314bMN11 | ‘M92-220 – Long’ | Bolon et al., 2014 | 16 Gy | M4 | GSE58172 | GSM1402664 |
| Fast Neutron  (No-Phenotype) | 3R27C90Cer315cMN11 | ‘M92-220 – Long’ | Bolon et al., 2014 | 16 Gy | M4 | GSE58172 | GSM1402665 |
| Fast Neutron  (No-Phenotype) | 3R33C61Ccr316aMN11 | ‘M92-220 – Long’ | Bolon et al., 2014 | 16 Gy | M4 | GSE58172 | GSM1402666 |
| Fast Neutron  (No-Phenotype) | 4R02C19Car317bMN11 | ‘M92-220 – Long’ | Bolon et al., 2014 | 16 Gy | M4 | GSE58172 | GSM1402670 |
| Fast Neutron  (No-Phenotype) | 4R03C13Cbr318cMN11 | ‘M92-220 – Long’ | Bolon et al., 2014 | 16 Gy | M4 | GSE58172 | GSM1402671 |
| Fast Neutron  (No-Phenotype) | 4R09C72Dar319cMN11 | ‘M92-220 – Long’ | Bolon et al., 2014 | 32 Gy | M4 | GSE58172 | GSM1402674 |
| Fast Neutron  (No-Phenotype) | 4R17C66Dbr320bMN11 | ‘M92-220 – Long’ | Bolon et al., 2014 | 32 Gy | M4 | GSE58172 | GSM1402676 |
| Fast Neutron  (No-Phenotype) | 4R38C67Cbr321cMN11 | ‘M92-220 – Long’ | Bolon et al., 2014 | 16 Gy | M4 | GSE58172 | GSM1402678 |
| Fast Neutron  (No-Phenotype) | 5R16C69Abr322aMN11 | ‘M92-220 – Long’ | Bolon et al., 2014 | 4 Gy | M4 | GSE58172 | GSM1402688 |
| Fast Neutron  (No-Phenotype) | 5R28C09Cdr323aMn11 | ‘M92-220 – Long’ | Bolon et al., 2014 | 16 Gy | M4 | GSE58172 | GSM1402689 |
| Fast Neutron  (No-Phenotype) | 5R29C21Chr324bMN11 | ‘M92-220 – Long’ | Bolon et al., 2014 | 16 Gy | M4 | GSE58172 | GSM1402690 |
| Fast Neutron (Mutant Phenotype) | FN 02 (FN0173217.03.09.01.M5) | ‘M92-220 – Long’ | Bolon et al., 2014 | 16 Gy | M5 | GSE58172 | GSM1402727 |
| Fast Neutron (Mutant Phenotype) | FN 03 (FN0172932.09.08.01.M5) | ‘M92-220 – Long’ | Bolon et al., 2014 | 16 Gy | M5 | GSE58172 | GSM1402726 |
| Fast Neutron (Mutant Phenotype) | FN 04 (FN0175143.05.06.01.M5) | ‘M92-220 – Long’ | Bolon et al., 2014 | 16 Gy | M5 | GSE58172 | GSM1402728 |
| Fast Neutron (Mutant Phenotype) | FN 05  (FN0171501.01.02.M4) | ‘M92-220 – Long’ | Bolon et al., 2014 | 32 Gy | M4 | GSE58172 | GSM1402724 |
| Fast Neutron (Mutant Phenotype) | FN 06  (FN0131633.06.01.M4) | ‘M92-220 – Long’ | Bolon et al., 2014 | 16 Gy | M4 | GSE58172 | GSM1402662 |
| Fast Neutron (Mutant Phenotype) | FN 07 (FN0112228.06.02.01.M5) | ‘M92-220 – Long’ | Bolon et al., 2014 | 16 Gy | M5 | GSE58172 | GSM1402588 |
| Fast Neutron (Mutant Phenotype) | FN 08 (FN0112885.02.06.03.M5) | ‘M92-220 – Long’ | Bolon et al., 2014 | 16 Gy | M5 | GSE58172 | GSM1402592 |
| Fast Neutron (Mutant Phenotype) | FN 09  (FN0163764.04.01.M4) | ‘M92-220 – Long’ | Bolon et al., 2014 | 32 Gy | M4 | GSE58172 | GSM1402700 |
| Fast Neutron (Mutant Phenotype) | FN 10 (FN0164160.03.02.01.01.M6) | ‘M92-220 – Long’ | Bolon et al., 2014 | 32 Gy | M6 | GSE58172 | GSM1402707 |
| Fast Neutron (Mutant Phenotype) | FN 11 (FN0175501.x2.02.01.M5) | ‘M92-220 – Long’ | Bolon et al., 2014 | 16 Gy | M5 | GSE58172 | GSM1402716 |

Table S6. Sequences of PCR primers used for genotyping.

| SV location and Background | Primer Name | Sequence | Backup Primer |
| --- | --- | --- | --- |
| Chromosome 1, WPT_384-1-1 | F_Deletion | AGTAGCGGAACTGGTGTGGT | TTTGTCATCCTCGTCGTTTG |
| Chromosome 1, WPT_384-1-1 | F_WildType | GTTTGTTGTGGAGTGTTAGC |  |
| Chromosome 1, WPT_384-1-1 | Reverse | CACAAAGGCCACAAATTGAA | CATGCACAACGTGGTCTTTC |
| Chromosome 11, WPT_389-2-2 | F_Deletion | CACAAACTTGGACTGCTGGA |  |
| Chromosome 11, WPT_389-2-2 | F_WildType | GGAGTGCAGGTTGCTTGAGC |  |
| Chromosome 11, WPT_389-2-2 | Reverse | TAGTTTTCGTCGGCAAAAGG |  |
| Chromosome 13, WPT_301-3-13 | F_Duplication | GCTCAATTTGGTCCTTTCCA |  |
| Chromosome 13, WPT_301-3-13 | F_WildType | GCATGAAAGGGTATAGGAAGG |  |
| Chromosome 13, WPT_301-3-13 | Reverse | GTCTAGAACCCTATCCGTGCAC |  |
| Chromosome 19, WPT_391-1-6 | F_Deletion | GTGTAGTAAGAAAATGCTCACC |  |
| Chromosome 19, WPT_391-1-6 | Reverse | GCCATCAATGCCTCAGAAAC |  |
