# Supplementary material for: Genomic variation and DNA repair associated with soybean transgenesis: a comparison to cultivars and mutagenized plants
Source: BMC Biotechnol. 2016 May 12;16:41. doi: 10.1186/s12896-016-0271-z (PMC4866027; doi:10.1186/s12896-016-0271-z)
Supplement: Additional file 2: Figure S1. — A novel deletion detected on chromosome 01 in transgenic plant WPT_384-1-1. Figure S2. A novel deletion on chromosome 19 in transgenic plant WPT_391-1-6. Figure S3. Test for intracultivar variation in the parental lines by genotyping 47 individuals taken from GRIN stocks of the varieties ‘Bert’ and ‘Williams 82’. Figure S4. Genotyping diverse lines including the 41 SoyNAM parents, cultivars ‘Archer’, ‘Minsoy’, and ‘Noir1’, ‘Bert-MN-01’, and ‘Wm82-ISU-01,’ for previous evidence of SV found in transformed plants. Figure S5. Novel deletion on chromosome 11 in transgenic plant WPT_389-2-2. Figure S6. Novel duplication on chromosome 13 in transgenic plant WPT_301-3-13. Figure S7. Southern blot analysis of HindIII digested genomic DNA. Figure S8. Microhomology of sequences at the T-DNA left border and the sites of genomic integration for three transgenic plants. Figure S9. Structure of the heterozygous transgene insertion on chromosome 05 in transgenic plant WPT_391-1-6. Figure S10. Transgene insertion on chromosome 13 in transgenic plant WPT_389-2-2. Figure S11. Pipeline for utilizing resequencing data in this study. (PPTX 3058 kb) [file 12896_2016_271_MOESM2_ESM.pptx]

## Slide 1
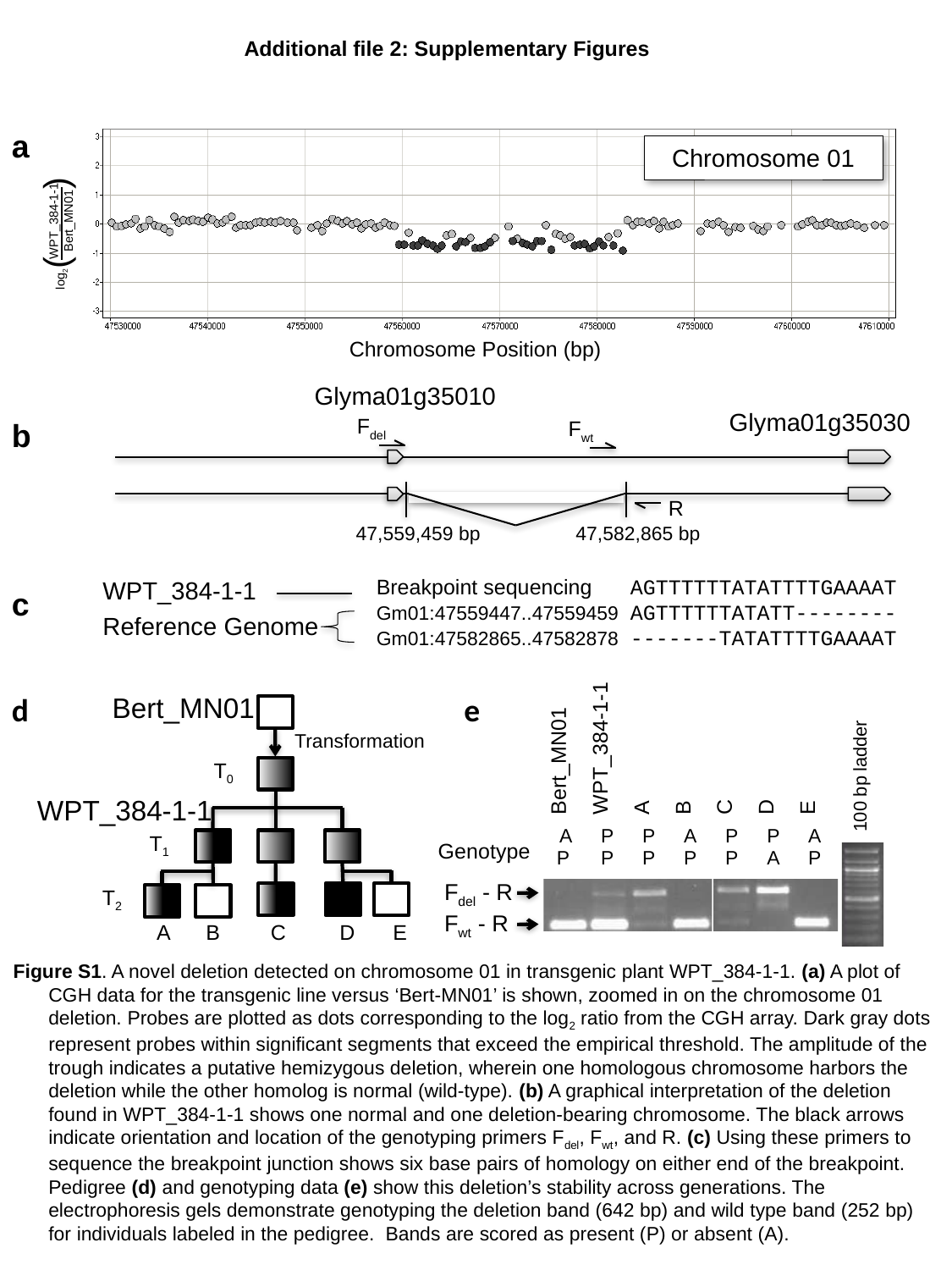

Additional file 2: Supplementary Figures
a
Chromosome 01
( )
WPT_384-1-1
Bert_MN01
log2
Chromosome Position (bp)
Glyma01g35010
Glyma01g35030
Fdel
b
Fwt
R
47,559,459 bp
47,582,865 bp
Breakpoint sequencing	AGTTTTTTATATTTTGAAAAT
Gm01:47559447..47559459	AGTTTTTTATATT--------
Gm01:47582865..47582878	-------TATATTTTGAAAAT
WPT_384-1-1
Reference Genome
c
| Bert\_MN01 | WPT\_384-1-1 | A | B | C | D | E |
| --- | --- | --- | --- | --- | --- | --- |
| A P | P P | P P | A P | P P | P A | A P |
e
Bert_MN01
d
Transformation
T0
100 bp ladder
WPT_384-1-1
T1
Genotype
Fdel - R
Fwt - R
T2
A
B
C
D
E
Figure S1. A novel deletion detected on chromosome 01 in transgenic plant WPT_384-1-1. (a) A plot of CGH data for the transgenic line versus ‘Bert-MN01’ is shown, zoomed in on the chromosome 01 deletion. Probes are plotted as dots corresponding to the log2 ratio from the CGH array. Dark gray dots represent probes within significant segments that exceed the empirical threshold. The amplitude of the trough indicates a putative hemizygous deletion, wherein one homologous chromosome harbors the deletion while the other homolog is normal (wild-type). (b) A graphical interpretation of the deletion found in WPT_384-1-1 shows one normal and one deletion-bearing chromosome. The black arrows indicate orientation and location of the genotyping primers Fdel, Fwt, and R. (c) Using these primers to sequence the breakpoint junction shows six base pairs of homology on either end of the breakpoint. Pedigree (d) and genotyping data (e) show this deletion’s stability across generations. The electrophoresis gels demonstrate genotyping the deletion band (642 bp) and wild type band (252 bp) for individuals labeled in the pedigree. Bands are scored as present (P) or absent (A).

## Slide 2
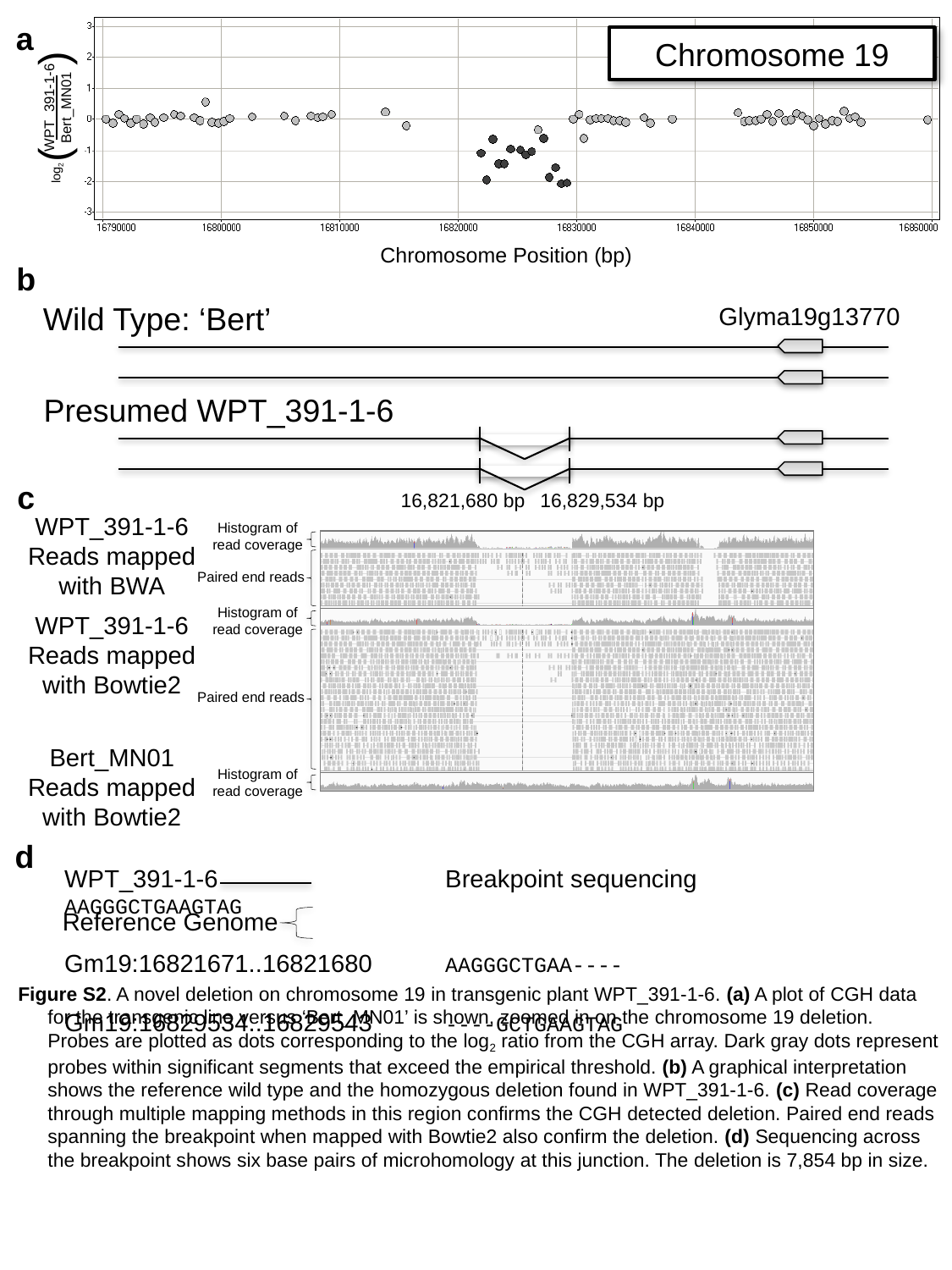

a
Chromosome 19
( )
WPT_391-1-6
Bert_MN01
log2
Chromosome Position (bp)
b
Wild Type: ‘Bert’
Glyma19g13770
Presumed WPT_391-1-6
c
16,821,680 bp
16,829,534 bp
WPT_391-1-6
Reads mapped with BWA
Histogram of read coverage
Paired end reads
Histogram of read coverage
WPT_391-1-6
Reads mapped with Bowtie2
Paired end reads
Bert_MN01 Reads mapped with Bowtie2
Histogram of read coverage
d
WPT_391-1-6		Breakpoint sequencing		AAGGGCTGAAGTAG
				Gm19:16821671..16821680	AAGGGCTGAA----
				Gm19:16829534..16829543	----GCTGAAGTAG
Reference Genome
 Figure S2. A novel deletion on chromosome 19 in transgenic plant WPT_391-1-6. (a) A plot of CGH data for the transgenic line versus ‘Bert_MN01’ is shown, zoomed in on the chromosome 19 deletion. Probes are plotted as dots corresponding to the log2 ratio from the CGH array. Dark gray dots represent probes within significant segments that exceed the empirical threshold. (b) A graphical interpretation shows the reference wild type and the homozygous deletion found in WPT_391-1-6. (c) Read coverage through multiple mapping methods in this region confirms the CGH detected deletion. Paired end reads spanning the breakpoint when mapped with Bowtie2 also confirm the deletion. (d) Sequencing across the breakpoint shows six base pairs of microhomology at this junction. The deletion is 7,854 bp in size.

## Slide 3
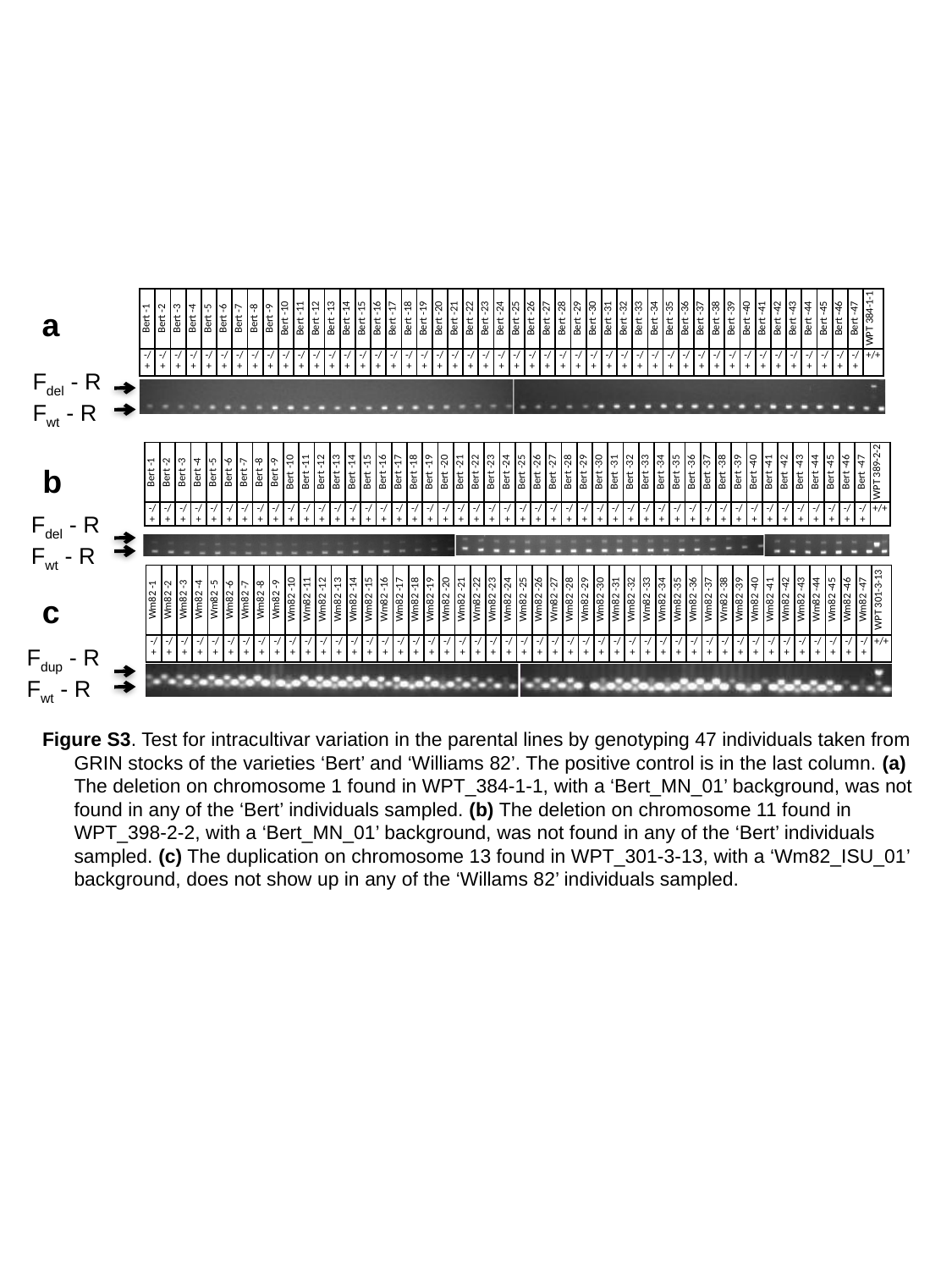

| Bert -1 | Bert -2 | Bert -3 | Bert -4 | Bert -5 | Bert -6 | Bert -7 | Bert -8 | Bert -9 | Bert -10 | Bert -11 | Bert -12 | Bert -13 | Bert -14 | Bert -15 | Bert -16 | Bert -17 | Bert -18 | Bert -19 | Bert -20 | Bert -21 | Bert -22 | Bert -23 | Bert -24 | Bert -25 | Bert -26 | Bert -27 | Bert -28 | Bert -29 | Bert -30 | Bert -31 | Bert -32 | Bert -33 | Bert -34 | Bert -35 | Bert -36 | Bert -37 | Bert -38 | Bert -39 | Bert -40 | Bert -41 | Bert -42 | Bert -43 | Bert -44 | Bert -45 | Bert -46 | Bert -47 | WPT 384-1-1 |
| --- | --- | --- | --- | --- | --- | --- | --- | --- | --- | --- | --- | --- | --- | --- | --- | --- | --- | --- | --- | --- | --- | --- | --- | --- | --- | --- | --- | --- | --- | --- | --- | --- | --- | --- | --- | --- | --- | --- | --- | --- | --- | --- | --- | --- | --- | --- | --- |
| -/+ | -/+ | -/+ | -/+ | -/+ | -/+ | -/+ | -/+ | -/+ | -/+ | -/+ | -/+ | -/+ | -/+ | -/+ | -/+ | -/+ | -/+ | -/+ | -/+ | -/+ | -/+ | -/+ | -/+ | -/+ | -/+ | -/+ | -/+ | -/+ | -/+ | -/+ | -/+ | -/+ | -/+ | -/+ | -/+ | -/+ | -/+ | -/+ | -/+ | -/+ | -/+ | -/+ | -/+ | -/+ | -/+ | -/+ | +/+ |
a
Fdel - R
Fwt - R
| Bert -1 | Bert -2 | Bert -3 | Bert -4 | Bert -5 | Bert -6 | Bert -7 | Bert -8 | Bert -9 | Bert -10 | Bert -11 | Bert -12 | Bert -13 | Bert -14 | Bert -15 | Bert -16 | Bert -17 | Bert -18 | Bert -19 | Bert -20 | Bert -21 | Bert -22 | Bert -23 | Bert -24 | Bert -25 | Bert -26 | Bert -27 | Bert -28 | Bert -29 | Bert -30 | Bert -31 | Bert -32 | Bert -33 | Bert -34 | Bert -35 | Bert -36 | Bert -37 | Bert -38 | Bert -39 | Bert -40 | Bert -41 | Bert -42 | Bert -43 | Bert -44 | Bert -45 | Bert -46 | Bert -47 | WPT 389-2-2 |
| --- | --- | --- | --- | --- | --- | --- | --- | --- | --- | --- | --- | --- | --- | --- | --- | --- | --- | --- | --- | --- | --- | --- | --- | --- | --- | --- | --- | --- | --- | --- | --- | --- | --- | --- | --- | --- | --- | --- | --- | --- | --- | --- | --- | --- | --- | --- | --- |
| -/+ | -/+ | -/+ | -/+ | -/+ | -/+ | -/+ | -/+ | -/+ | -/+ | -/+ | -/+ | -/+ | -/+ | -/+ | -/+ | -/+ | -/+ | -/+ | -/+ | -/+ | -/+ | -/+ | -/+ | -/+ | -/+ | -/+ | -/+ | -/+ | -/+ | -/+ | -/+ | -/+ | -/+ | -/+ | -/+ | -/+ | -/+ | -/+ | -/+ | -/+ | -/+ | -/+ | -/+ | -/+ | -/+ | -/+ | +/+ |
b
Fdel - R
Fwt - R
| Wm82 -1 | Wm82 -2 | Wm82 -3 | Wm82 -4 | Wm82 -5 | Wm82 -6 | Wm82 -7 | Wm82 -8 | Wm82 -9 | Wm82 -10 | Wm82 -11 | Wm82 -12 | Wm82 -13 | Wm82 -14 | Wm82 -15 | Wm82 -16 | Wm82 -17 | Wm82 -18 | Wm82 -19 | Wm82 -20 | Wm82 -21 | Wm82 -22 | Wm82 -23 | Wm82 -24 | Wm82 -25 | Wm82 -26 | Wm82 -27 | Wm82 -28 | Wm82 -29 | Wm82 -30 | Wm82 -31 | Wm82 -32 | Wm82 -33 | Wm82 -34 | Wm82 -35 | Wm82 -36 | Wm82 -37 | Wm82 -38 | Wm82 -39 | Wm82 -40 | Wm82 -41 | Wm82 -42 | Wm82 -43 | Wm82 -44 | Wm82 -45 | Wm82 -46 | Wm82 -47 | WPT 301-3-13 |
| --- | --- | --- | --- | --- | --- | --- | --- | --- | --- | --- | --- | --- | --- | --- | --- | --- | --- | --- | --- | --- | --- | --- | --- | --- | --- | --- | --- | --- | --- | --- | --- | --- | --- | --- | --- | --- | --- | --- | --- | --- | --- | --- | --- | --- | --- | --- | --- |
| -/+ | -/+ | -/+ | -/+ | -/+ | -/+ | -/+ | -/+ | -/+ | -/+ | -/+ | -/+ | -/+ | -/+ | -/+ | -/+ | -/+ | -/+ | -/+ | -/+ | -/+ | -/+ | -/+ | -/+ | -/+ | -/+ | -/+ | -/+ | -/+ | -/+ | -/+ | -/+ | -/+ | -/+ | -/+ | -/+ | -/+ | -/+ | -/+ | -/+ | -/+ | -/+ | -/+ | -/+ | -/+ | -/+ | -/+ | +/+ |
c
Fdup - R
Fwt - R
Figure S3. Test for intracultivar variation in the parental lines by genotyping 47 individuals taken from GRIN stocks of the varieties ‘Bert’ and ‘Williams 82’. The positive control is in the last column. (a) The deletion on chromosome 1 found in WPT_384-1-1, with a ‘Bert_MN_01’ background, was not found in any of the ‘Bert’ individuals sampled. (b) The deletion on chromosome 11 found in WPT_398-2-2, with a ‘Bert_MN_01’ background, was not found in any of the ‘Bert’ individuals sampled. (c) The duplication on chromosome 13 found in WPT_301-3-13, with a ‘Wm82_ISU_01’ background, does not show up in any of the ‘Willams 82’ individuals sampled.

## Slide 4
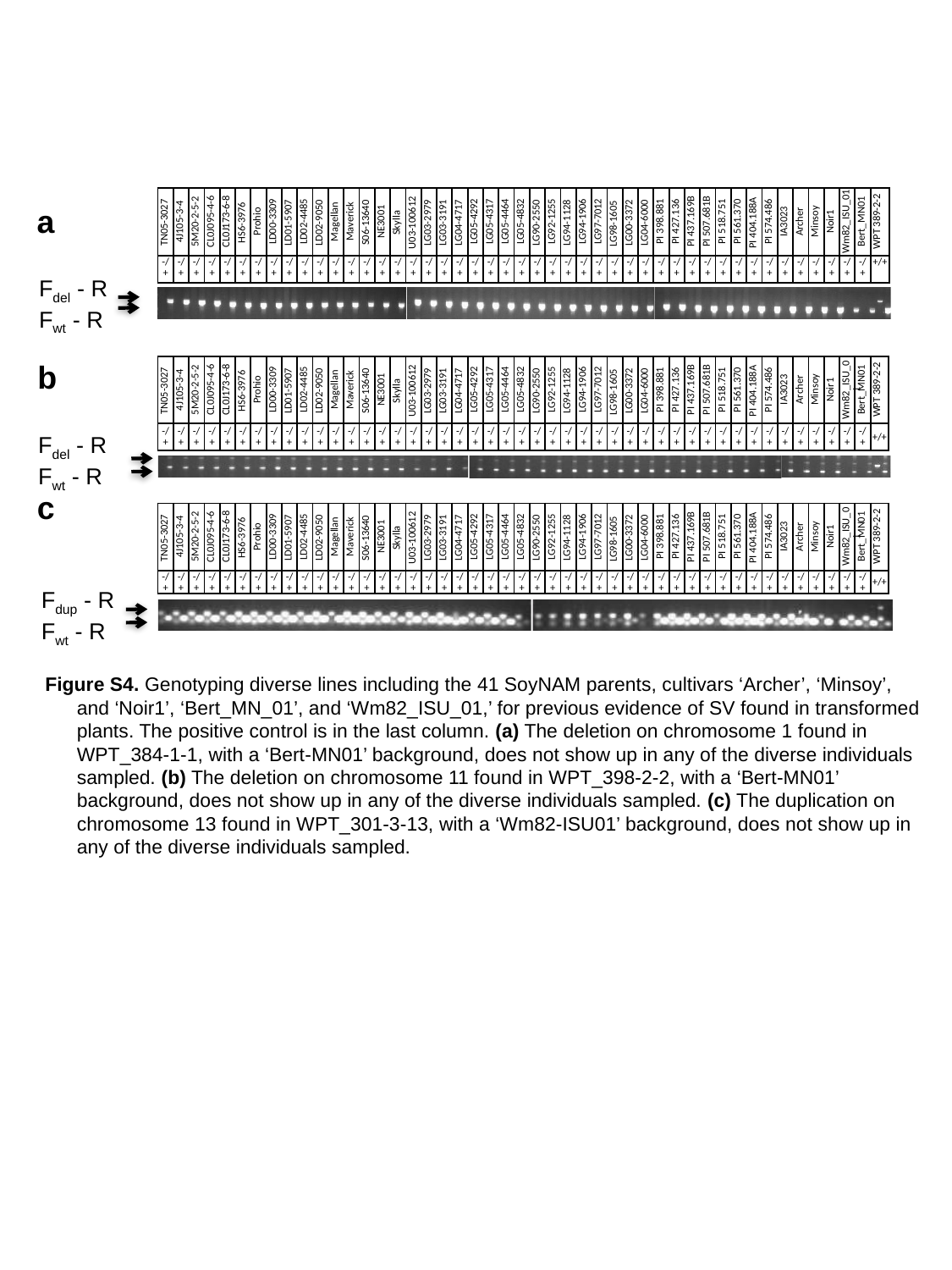

| TN05-3027 | 4J105-3-4 | 5M20-2-5-2 | CL0J095-4-6 | CL0J173-6-8 | HS6-3976 | Prohio | LD00-3309 | LD01-5907 | LD02-4485 | LD02-9050 | Magellan | Maverick | S06-13640 | NE3001 | Skylla | U03-100612 | LG03-2979 | LG03-3191 | LG04-4717 | LG05-4292 | LG05-4317 | LG05-4464 | LG05-4832 | LG90-2550 | LG92-1255 | LG94-1128 | LG94-1906 | LG97-7012 | LG98-1605 | LG00-3372 | LG04-6000 | PI 398.881 | PI 427.136 | PI 437.169B | PI 507.681B | PI 518.751 | PI 561.370 | PI 404.188A | PI 574.486 | IA3023 | Archer | Minsoy | Noir1 | Wm82\_ISU\_01 | Bert\_MN01 | WPT 389-2-2 |
| --- | --- | --- | --- | --- | --- | --- | --- | --- | --- | --- | --- | --- | --- | --- | --- | --- | --- | --- | --- | --- | --- | --- | --- | --- | --- | --- | --- | --- | --- | --- | --- | --- | --- | --- | --- | --- | --- | --- | --- | --- | --- | --- | --- | --- | --- | --- |
| -/+ | -/+ | -/+ | -/+ | -/+ | -/+ | -/+ | -/+ | -/+ | -/+ | -/+ | -/+ | -/+ | -/+ | -/+ | -/+ | -/+ | -/+ | -/+ | -/+ | -/+ | -/+ | -/+ | -/+ | -/+ | -/+ | -/+ | -/+ | -/+ | -/+ | -/+ | -/+ | -/+ | -/+ | -/+ | -/+ | -/+ | -/+ | -/+ | -/+ | -/+ | -/+ | -/+ | -/+ | -/+ | -/+ | +/+ |
a
Fdel - R
Fwt - R
b
| TN05-3027 | 4J105-3-4 | 5M20-2-5-2 | CL0J095-4-6 | CL0J173-6-8 | HS6-3976 | Prohio | LD00-3309 | LD01-5907 | LD02-4485 | LD02-9050 | Magellan | Maverick | S06-13640 | NE3001 | Skylla | U03-100612 | LG03-2979 | LG03-3191 | LG04-4717 | LG05-4292 | LG05-4317 | LG05-4464 | LG05-4832 | LG90-2550 | LG92-1255 | LG94-1128 | LG94-1906 | LG97-7012 | LG98-1605 | LG00-3372 | LG04-6000 | PI 398.881 | PI 427.136 | PI 437.169B | PI 507.681B | PI 518.751 | PI 561.370 | PI 404.188A | PI 574.486 | IA3023 | Archer | Minsoy | Noir1 | Wm82\_ISU\_01 | Bert\_MN01 | WPT 389-2-2 |
| --- | --- | --- | --- | --- | --- | --- | --- | --- | --- | --- | --- | --- | --- | --- | --- | --- | --- | --- | --- | --- | --- | --- | --- | --- | --- | --- | --- | --- | --- | --- | --- | --- | --- | --- | --- | --- | --- | --- | --- | --- | --- | --- | --- | --- | --- | --- |
| -/+ | -/+ | -/+ | -/+ | -/+ | -/+ | -/+ | -/+ | -/+ | -/+ | -/+ | -/+ | -/+ | -/+ | -/+ | -/+ | -/+ | -/+ | -/+ | -/+ | -/+ | -/+ | -/+ | -/+ | -/+ | -/+ | -/+ | -/+ | -/+ | -/+ | -/+ | -/+ | -/+ | -/+ | -/+ | -/+ | -/+ | -/+ | -/+ | -/+ | -/+ | -/+ | -/+ | -/+ | -/+ | -/+ | +/+ |
Fdel - R
Fwt - R
c
| TN05-3027 | 4J105-3-4 | 5M20-2-5-2 | CL0J095-4-6 | CL0J173-6-8 | HS6-3976 | Prohio | LD00-3309 | LD01-5907 | LD02-4485 | LD02-9050 | Magellan | Maverick | S06-13640 | NE3001 | Skylla | U03-100612 | LG03-2979 | LG03-3191 | LG04-4717 | LG05-4292 | LG05-4317 | LG05-4464 | LG05-4832 | LG90-2550 | LG92-1255 | LG94-1128 | LG94-1906 | LG97-7012 | LG98-1605 | LG00-3372 | LG04-6000 | PI 398.881 | PI 427.136 | PI 437.169B | PI 507.681B | PI 518.751 | PI 561.370 | PI 404.188A | PI 574.486 | IA3023 | Archer | Minsoy | Noir1 | Wm82\_ISU\_01 | Bert\_MN01 | WPT 389-2-2 |
| --- | --- | --- | --- | --- | --- | --- | --- | --- | --- | --- | --- | --- | --- | --- | --- | --- | --- | --- | --- | --- | --- | --- | --- | --- | --- | --- | --- | --- | --- | --- | --- | --- | --- | --- | --- | --- | --- | --- | --- | --- | --- | --- | --- | --- | --- | --- |
| -/+ | -/+ | -/+ | -/+ | -/+ | -/+ | -/+ | -/+ | -/+ | -/+ | -/+ | -/+ | -/+ | -/+ | -/+ | -/+ | -/+ | -/+ | -/+ | -/+ | -/+ | -/+ | -/+ | -/+ | -/+ | -/+ | -/+ | -/+ | -/+ | -/+ | -/+ | -/+ | -/+ | -/+ | -/+ | -/+ | -/+ | -/+ | -/+ | -/+ | -/+ | -/+ | -/+ | -/+ | -/+ | -/+ | +/+ |
Fdup - R
Fwt - R
Figure S4. Genotyping diverse lines including the 41 SoyNAM parents, cultivars ‘Archer’, ‘Minsoy’, and ‘Noir1’, ‘Bert_MN_01’, and ‘Wm82_ISU_01,’ for previous evidence of SV found in transformed plants. The positive control is in the last column. (a) The deletion on chromosome 1 found in WPT_384-1-1, with a ‘Bert-MN01’ background, does not show up in any of the diverse individuals sampled. (b) The deletion on chromosome 11 found in WPT_398-2-2, with a ‘Bert-MN01’ background, does not show up in any of the diverse individuals sampled. (c) The duplication on chromosome 13 found in WPT_301-3-13, with a ‘Wm82-ISU01’ background, does not show up in any of the diverse individuals sampled.

## Slide 5
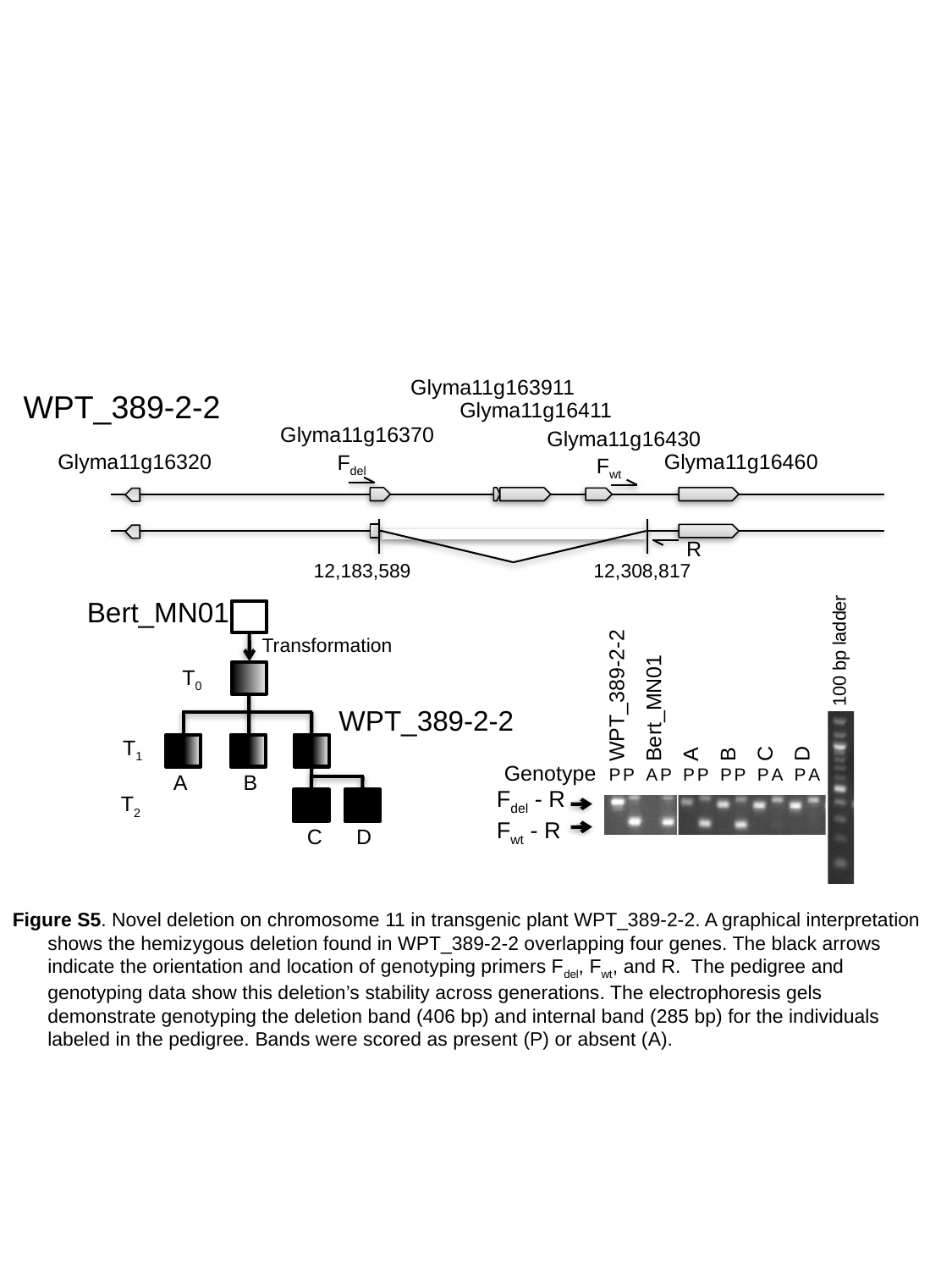

Glyma11g163911
WPT_389-2-2
Glyma11g16411
Glyma11g16370
Glyma11g16430
Glyma11g16460
Glyma11g16320
Fdel
Fwt
R
12,183,589
12,308,817
Bert_MN01
Transformation
T0
WPT_389-2-2
T1
B
A
T2
D
C
| WPT\_389-2-2 | Bert\_MN01 | A | B | C | D |
| --- | --- | --- | --- | --- | --- |
| P P | A P | P P | P P | P A | P A |
100 bp ladder
Genotype
Fdel - R
Fwt - R
Figure S5. Novel deletion on chromosome 11 in transgenic plant WPT_389-2-2. A graphical interpretation shows the hemizygous deletion found in WPT_389-2-2 overlapping four genes. The black arrows indicate the orientation and location of genotyping primers Fdel, Fwt, and R. The pedigree and genotyping data show this deletion’s stability across generations. The electrophoresis gels demonstrate genotyping the deletion band (406 bp) and internal band (285 bp) for the individuals labeled in the pedigree. Bands were scored as present (P) or absent (A).

## Slide 6
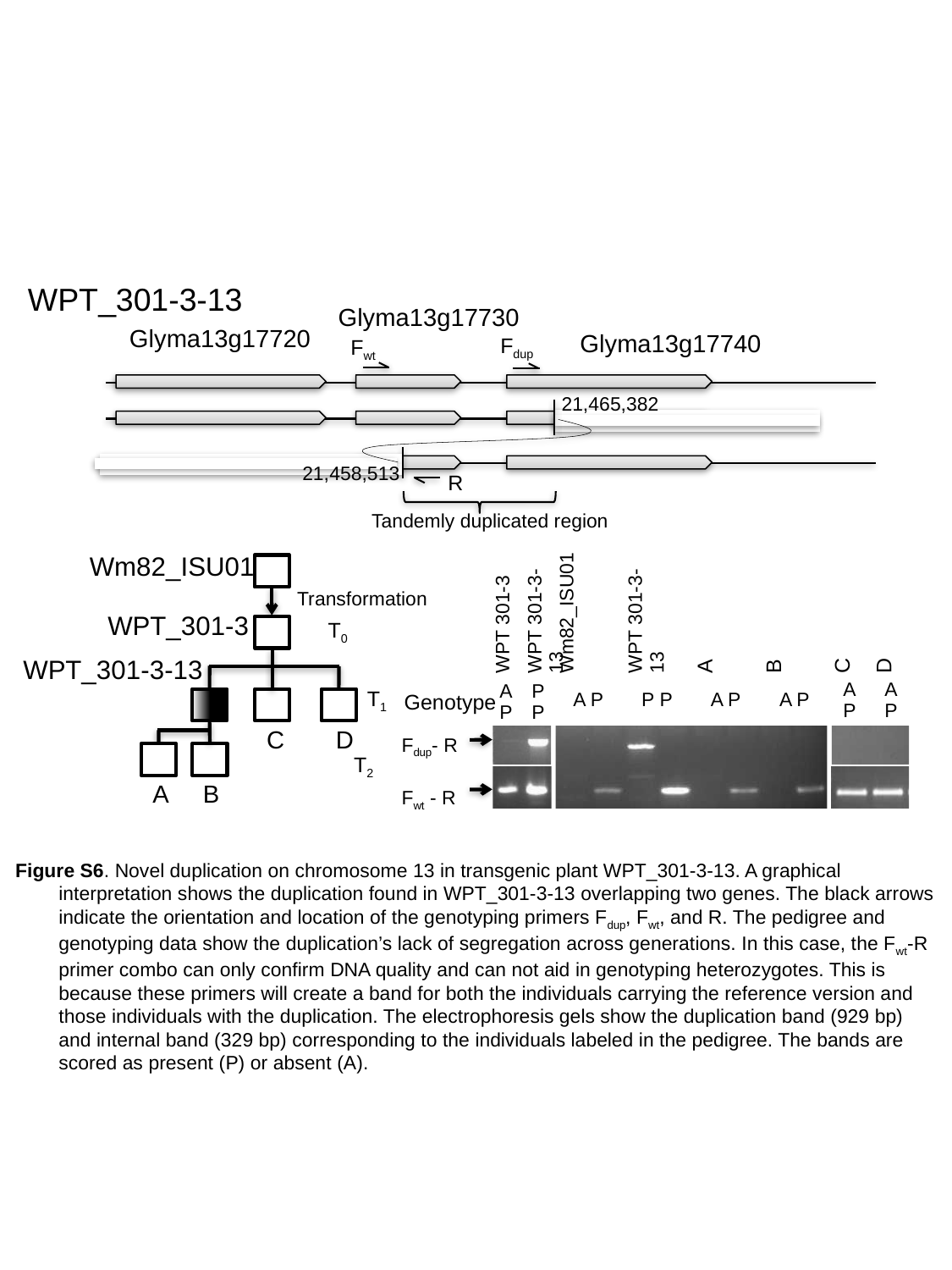

WPT_301-3-13
Glyma13g17730
Glyma13g17720
Glyma13g17740
Fdup
Fwt
21,465,382
21,458,513
R
Tandemly duplicated region
Wm82_ISU01
| WPT 301-3 | WPT 301-3-13 | Wm82\_ISU01 | WPT 301-3-13 | A | B | C | D |
| --- | --- | --- | --- | --- | --- | --- | --- |
| A P | P P | A P | P P | A P | A P | A P | A P |
Transformation
WPT_301-3
T0
WPT_301-3-13
T1
Genotype
C
D
Fdup- R
Fwt - R
T2
A
B
Figure S6. Novel duplication on chromosome 13 in transgenic plant WPT_301-3-13. A graphical interpretation shows the duplication found in WPT_301-3-13 overlapping two genes. The black arrows indicate the orientation and location of the genotyping primers Fdup, Fwt, and R. The pedigree and genotyping data show the duplication’s lack of segregation across generations. In this case, the Fwt-R primer combo can only confirm DNA quality and can not aid in genotyping heterozygotes. This is because these primers will create a band for both the individuals carrying the reference version and those individuals with the duplication. The electrophoresis gels show the duplication band (929 bp) and internal band (329 bp) corresponding to the individuals labeled in the pedigree. The bands are scored as present (P) or absent (A).

## Slide 7
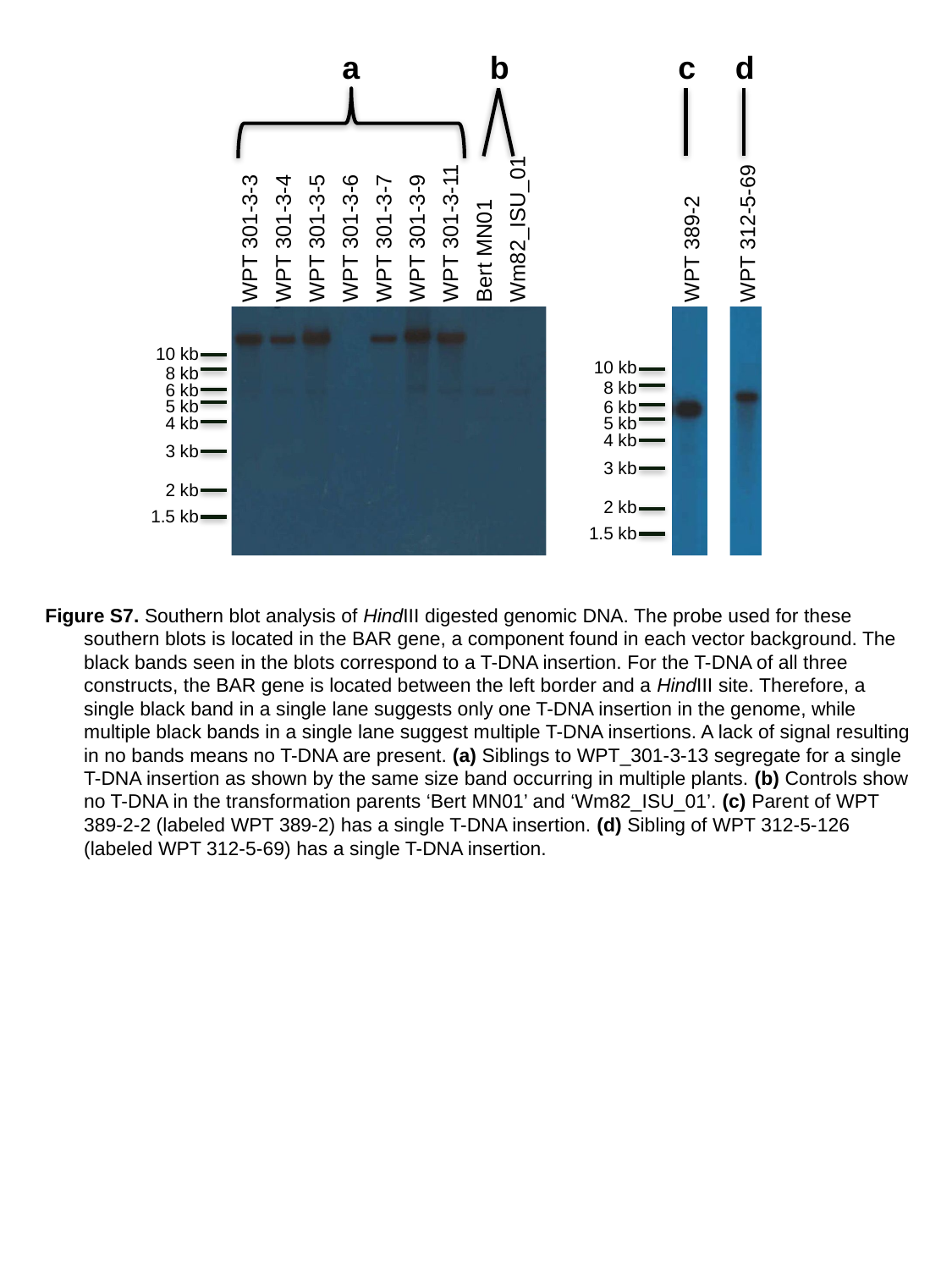

a
b
c
d
WPT 301-3-3
WPT 301-3-4
WPT 301-3-5
WPT 301-3-6
WPT 301-3-7
WPT 301-3-9
WPT 301-3-11
Bert MN01
Wm82_ISU_01
WPT 389-2
WPT 312-5-69
10 kb
10 kb
8 kb
8 kb
6 kb
5 kb
6 kb
4 kb
5 kb
4 kb
3 kb
3 kb
2 kb
2 kb
1.5 kb
1.5 kb
Figure S7. Southern blot analysis of HindIII digested genomic DNA. The probe used for these southern blots is located in the BAR gene, a component found in each vector background. The black bands seen in the blots correspond to a T-DNA insertion. For the T-DNA of all three constructs, the BAR gene is located between the left border and a HindIII site. Therefore, a single black band in a single lane suggests only one T-DNA insertion in the genome, while multiple black bands in a single lane suggest multiple T-DNA insertions. A lack of signal resulting in no bands means no T-DNA are present. (a) Siblings to WPT_301-3-13 segregate for a single T-DNA insertion as shown by the same size band occurring in multiple plants. (b) Controls show no T-DNA in the transformation parents ‘Bert MN01’ and ‘Wm82_ISU_01’. (c) Parent of WPT 389-2-2 (labeled WPT 389-2) has a single T-DNA insertion. (d) Sibling of WPT 312-5-126 (labeled WPT 312-5-69) has a single T-DNA insertion.

## Slide 8
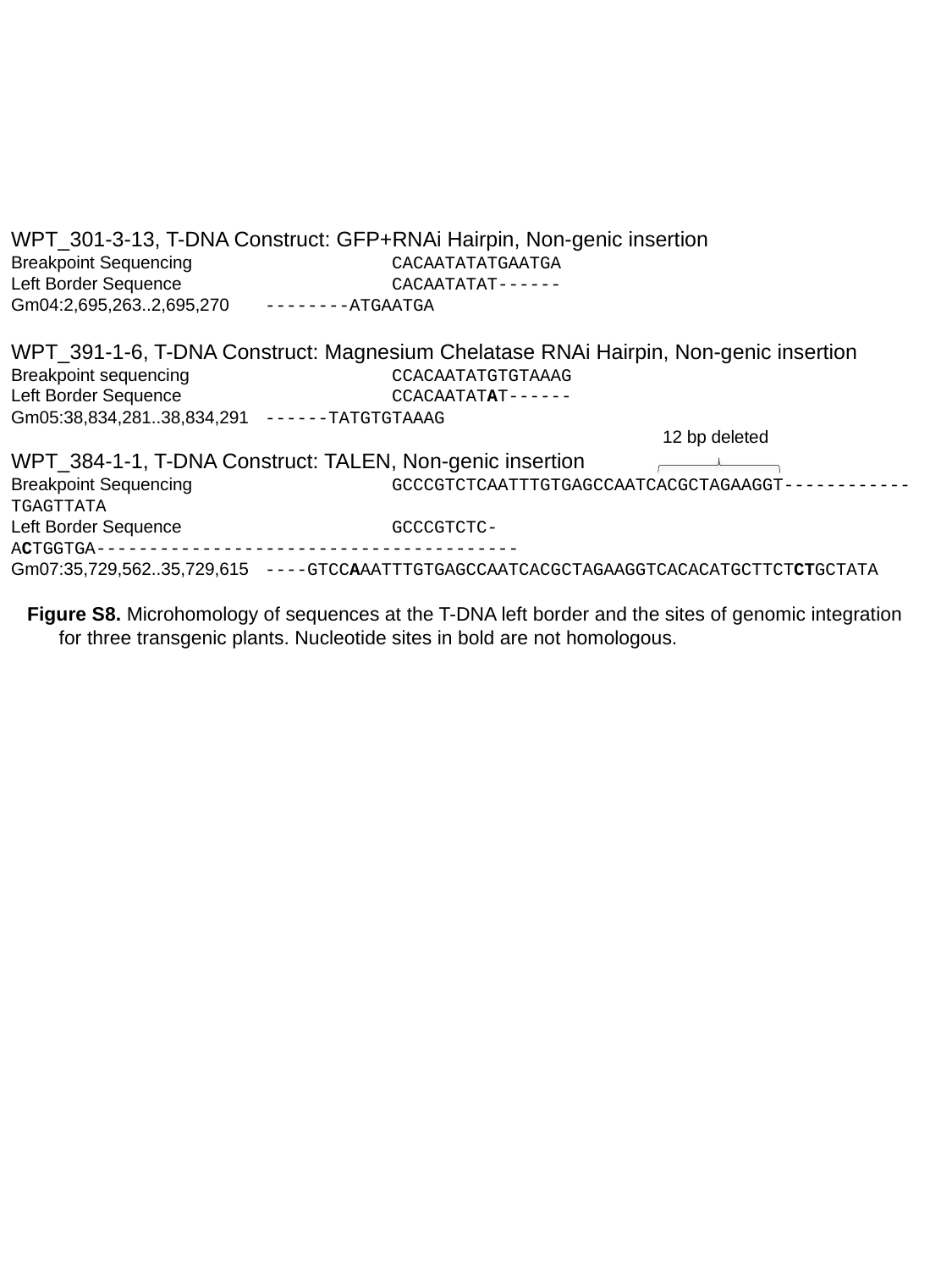

WPT_301-3-13, T-DNA Construct: GFP+RNAi Hairpin, Non-genic insertion
Breakpoint Sequencing 		CACAATATATGAATGA
Left Border Sequence		CACAATATAT------
Gm04:2,695,263..2,695,270	--------ATGAATGA
WPT_391-1-6, T-DNA Construct: Magnesium Chelatase RNAi Hairpin, Non-genic insertion
Breakpoint sequencing		CCACAATATGTGTAAAG
Left Border Sequence		CCACAATATAT------
Gm05:38,834,281..38,834,291	------TATGTGTAAAG
WPT_384-1-1, T-DNA Construct: TALEN, Non-genic insertion
Breakpoint Sequencing 		GCCCGTCTCAATTTGTGAGCCAATCACGCTAGAAGGT------------TGAGTTATA
Left Border Sequence		GCCCGTCTC-ACTGGTGA----------------------------------------
Gm07:35,729,562..35,729,615	----GTCCAAATTTGTGAGCCAATCACGCTAGAAGGTCACACATGCTTCTCTGCTATA
12 bp deleted
Figure S8. Microhomology of sequences at the T-DNA left border and the sites of genomic integration for three transgenic plants. Nucleotide sites in bold are not homologous.

## Slide 9
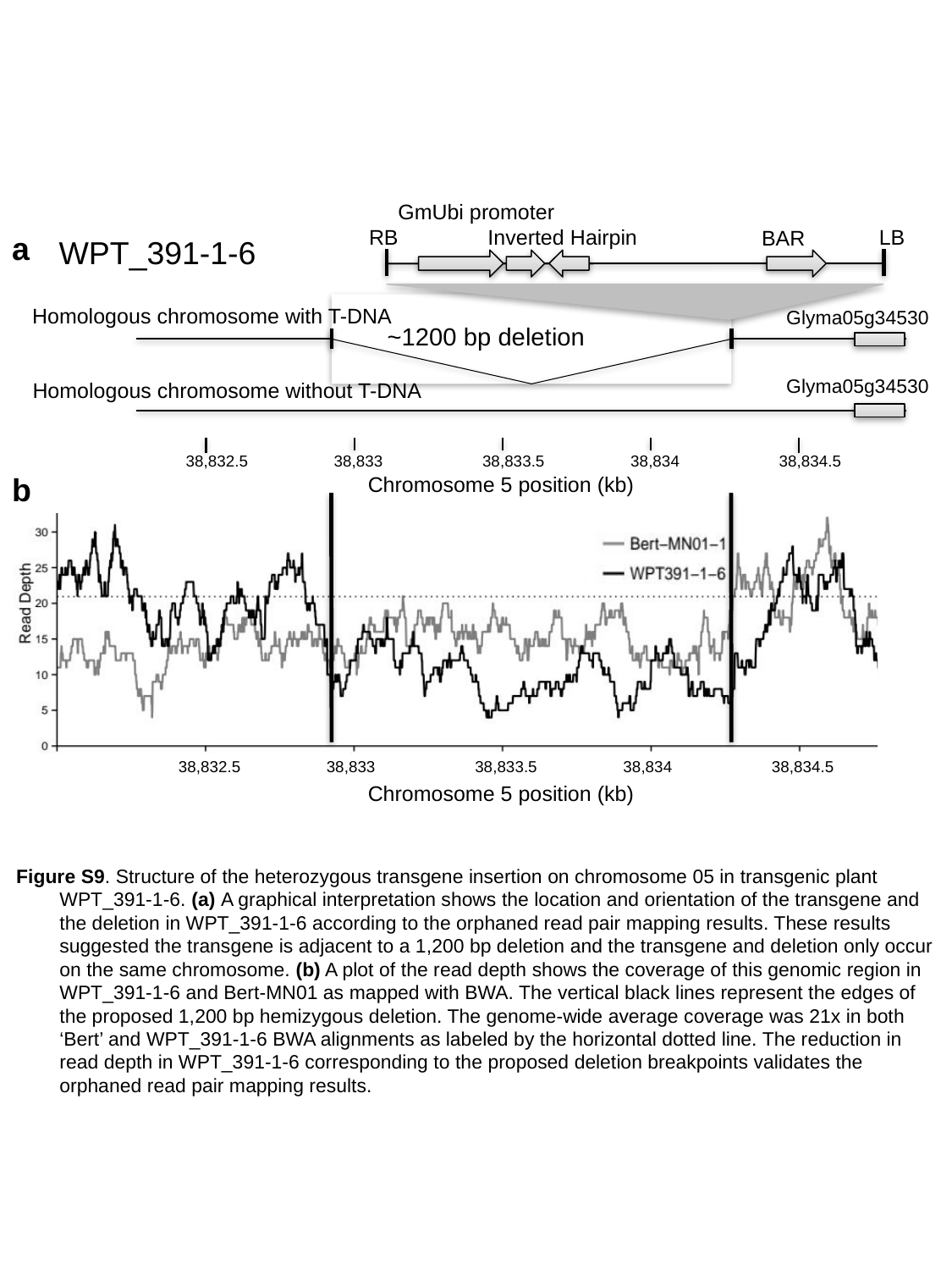

GmUbi promoter
Inverted Hairpin
RB
LB
BAR
a
WPT_391-1-6
Homologous chromosome with T-DNA
Glyma05g34530
~1200 bp deletion
Glyma05g34530
Homologous chromosome without T-DNA
38,832.5
38,833
38,833.5
38,834
38,834.5
b
Chromosome 5 position (kb)
38,832.5
38,833
38,833.5
38,834
38,834.5
Chromosome 5 position (kb)
Figure S9. Structure of the heterozygous transgene insertion on chromosome 05 in transgenic plant WPT_391-1-6. (a) A graphical interpretation shows the location and orientation of the transgene and the deletion in WPT_391-1-6 according to the orphaned read pair mapping results. These results suggested the transgene is adjacent to a 1,200 bp deletion and the transgene and deletion only occur on the same chromosome. (b) A plot of the read depth shows the coverage of this genomic region in WPT_391-1-6 and Bert-MN01 as mapped with BWA. The vertical black lines represent the edges of the proposed 1,200 bp hemizygous deletion. The genome-wide average coverage was 21x in both ‘Bert’ and WPT_391-1-6 BWA alignments as labeled by the horizontal dotted line. The reduction in read depth in WPT_391-1-6 corresponding to the proposed deletion breakpoints validates the orphaned read pair mapping results.

## Slide 10
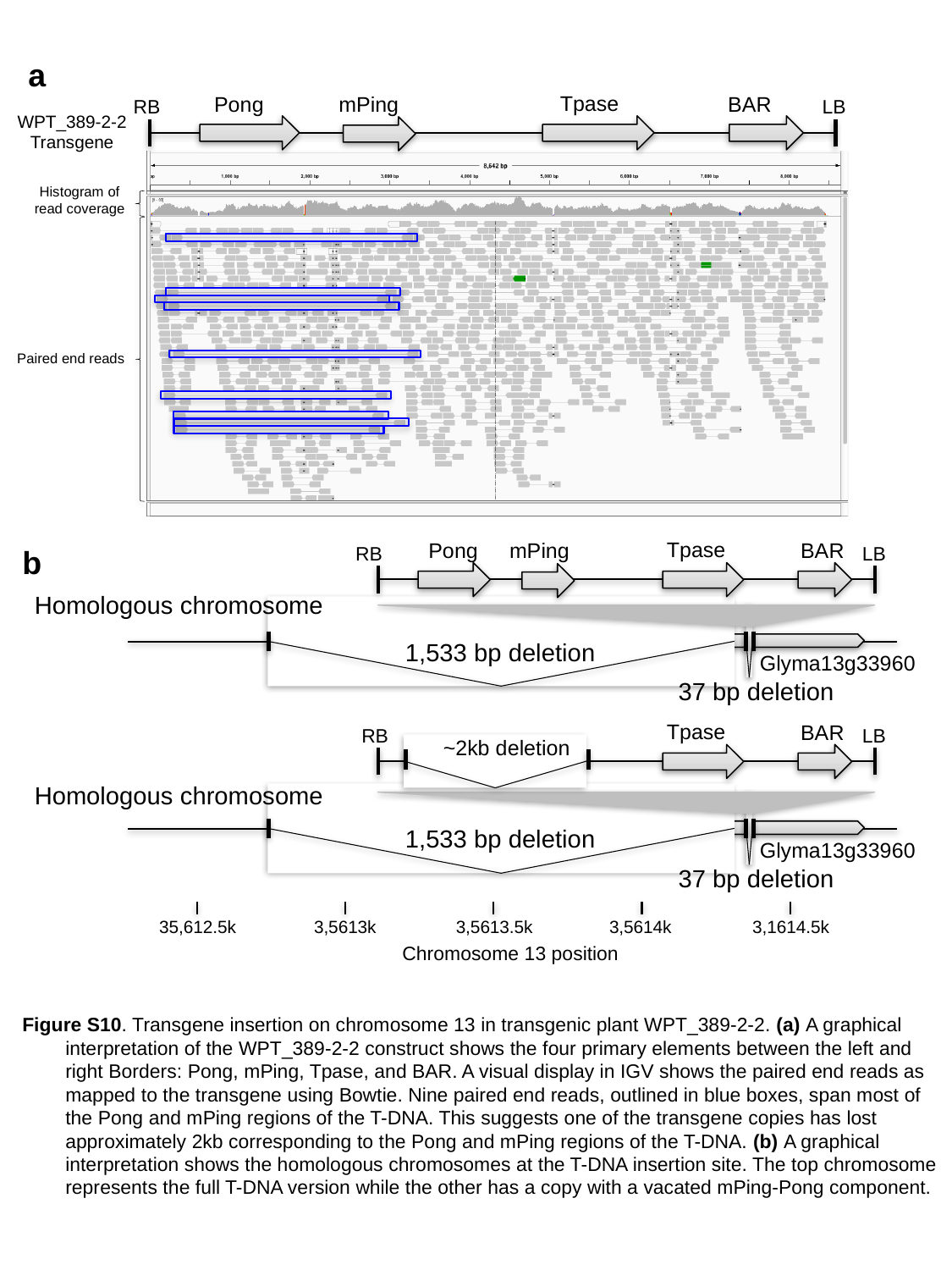

a
Tpase
Pong
mPing
BAR
RB
LB
WPT_389-2-2
Transgene
Histogram of read coverage
Paired end reads
Tpase
Pong
mPing
BAR
RB
LB
b
Homologous chromosome
1,533 bp deletion
Glyma13g33960
37 bp deletion
Tpase
BAR
RB
LB
~2kb deletion
Homologous chromosome
1,533 bp deletion
Glyma13g33960
37 bp deletion
35,612.5k
3,5613k
3,5613.5k
3,5614k
3,1614.5k
Chromosome 13 position
Figure S10. Transgene insertion on chromosome 13 in transgenic plant WPT_389-2-2. (a) A graphical interpretation of the WPT_389-2-2 construct shows the four primary elements between the left and right Borders: Pong, mPing, Tpase, and BAR. A visual display in IGV shows the paired end reads as mapped to the transgene using Bowtie. Nine paired end reads, outlined in blue boxes, span most of the Pong and mPing regions of the T-DNA. This suggests one of the transgene copies has lost approximately 2kb corresponding to the Pong and mPing regions of the T-DNA. (b) A graphical interpretation shows the homologous chromosomes at the T-DNA insertion site. The top chromosome represents the full T-DNA version while the other has a copy with a vacated mPing-Pong component.

## Slide 11
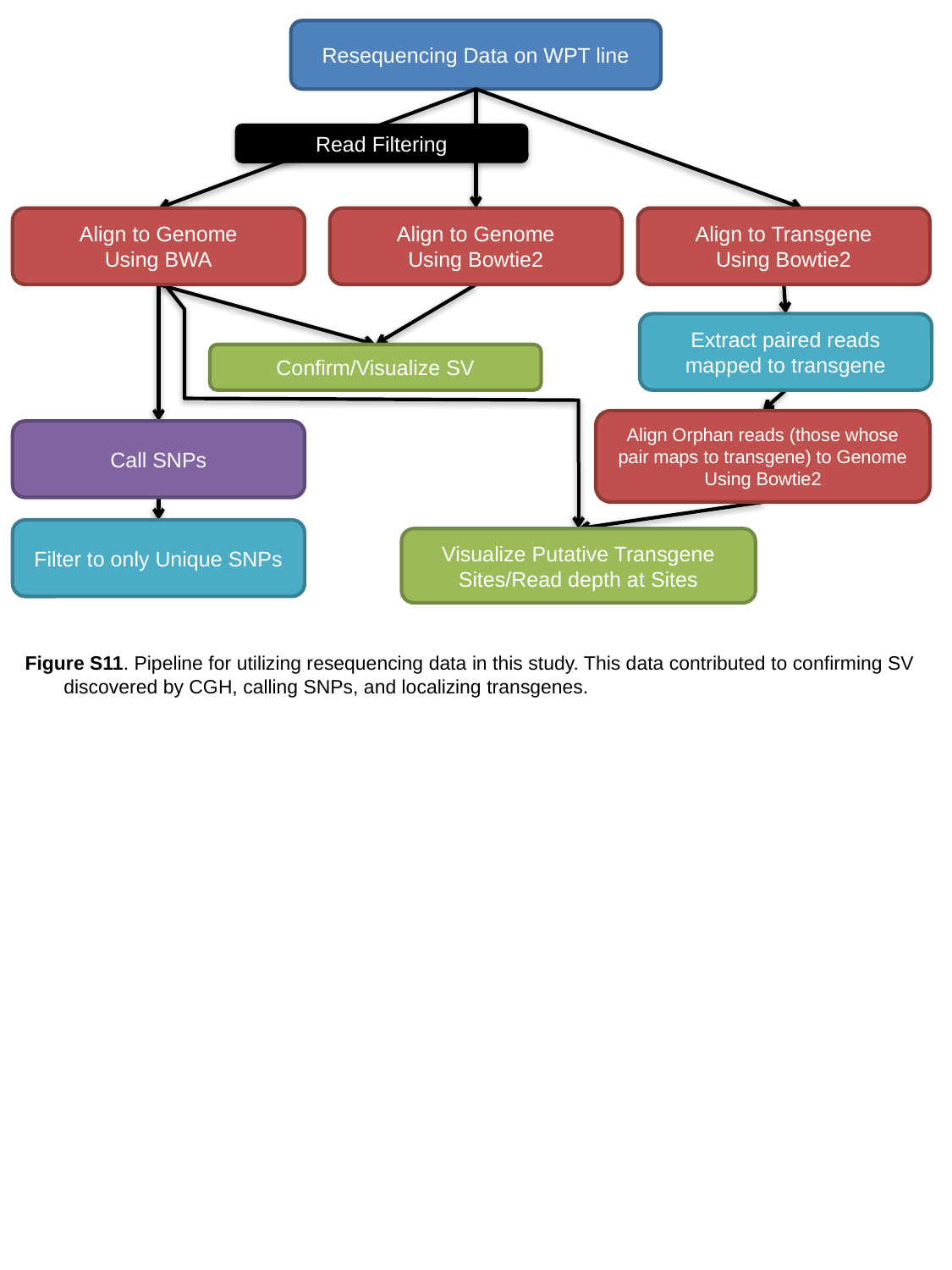

Resequencing Data on WPT line
Read Filtering
Align to Genome
Using BWA
Align to Genome
Using Bowtie2
Align to Transgene
Using Bowtie2
Extract paired reads mapped to transgene
Confirm/Visualize SV
Align Orphan reads (those whose pair maps to transgene) to Genome
Using Bowtie2
Call SNPs
Filter to only Unique SNPs
Visualize Putative Transgene Sites/Read depth at Sites
Figure S11. Pipeline for utilizing resequencing data in this study. This data contributed to confirming SV discovered by CGH, calling SNPs, and localizing transgenes.
